# Gut microbiome in PCOS associates to serum metabolomics: a cross-sectional study

**DOI:** 10.1038/s41598-022-25041-4

**Published:** 2022-12-23

**Authors:** Zheng Yu, Erqi Qin, Shirui Cheng, Han Yang, Rui Liu, Tian Xu, Yanqin Liu, Jing Yuan, Shuguang Yu, Jie Yang, Fanrong Liang

**Affiliations:** 1 College of Medical Information and Engineering, Chengdu University of Traditional Chinese Medicine, Chengdu, China; 2 Acupuncture Department, Chengdu Pidu District Hospital of Traditional Chinese Medicine, Chengdu, China; 3 Acupuncture and Tuina School, Chengdu University of Traditional Chinese Medicine, No. 37 Shi’er Qiao Rd, Chengdu, 610075 Sichuan China; 4 Graduate Program in Genetics, Genomics, and Bioinformatics, University of California, Riverside, CA USA; 5 Undergraduate Program in Department of Biochemistry, College of Agriculture and Life Science, Virginia Tech, Blacksburg, VA USA; 6 Gynecology Department, Chengdu Pidu District Hospital of Traditional Chinese Medicine, Chengdu, China

**Keywords:** Endocrine reproductive disorders, Predictive markers

## Abstract

The association between gut microbiome and chronic metabolic disease including polycystic ovary syndrome (PCOS), is well documented, however, the relationship between the gut microbiota and serum metabolites remains unknown. In this study, untargeted metabolomics together with a 16S rRNA gene sequencing tool was used to detect small molecule serum metabolites and the gut microbiome. We identified 15 differential metabolites between PCOS patients and the healthy control. Lysophosphatidylcholine (LPC) (18:2, 20:3, 18:1, P-16:0, 17:0, 15:0, 18:3, 20:4), phosphatidylcholine(PC), ganglioside GA2 (d18:1/16:0) and 1-linoleoylglycerophosphocholine were increased in the PCOS group, and the concentrations of phosphoniodidous acid, bilirubin, nicotinate beta-d-ribonucleotide and citric acid were decreased in the PCOS group, suggesting a lipid metabolism and energy metabolism disorder in the PCOS patients. The diversity of gut microbiota in PCOS group was lower than that in healthy controls. *Escherichia/Shigella*, *Alistipes* and an unnamed strain *0319_6G20* belonging to *Proteobacteria* were important distinguishing genera (LDA > 3.5) in PCOS. *Prevotella_9* was positively correlated with phosphoniodidous acid, nicotinate beta-d-ribonucleotide and citric acid concentrations, and negatively correlated with the concentration of LPC (20:3) and 1-linoleoylglycerophosphocholine; *Roseburia* was negatively correlated with LPC concentration (20:4), while the characteristic genus *0319_6G20* of PCOS was positively correlated with LPC concentration (20:3) (COR > 0.45). SF-36 in the PCOS group was significantly lower than that in the healthy control (HC) group, which was associated with the presence of *Escherichia-Shigella* and *Alistipes*. Our finding demonstrated the correlation between the gut microbiota and serum metabolites in PCOS, and therefore characteristic gut microbiota and metabolites may play an important role in the insulin resistance and the mood changes of PCOS patients.

## Introduction

Polycystic Ovary Syndrome (PCOS) occurs mostly in women of reproductive age. It is a complex syndrome characterized by excessive androgen, ovulation dysfunction, and organic polycystic ovary. Most patients also suffer from insulin resistance and lipid metabolism disorders^1^. It has been estimated that the clinical morbidity of PCOS in women of reproductive age worldwide is as high as 10%. According to the diagnostic criteria of the AE-PCOS Society and Rotterdam, the incidence can be as high as 15.3% and 19.9%, respectively^2^. PCOS can cause irregular menstrual cycles, infertility, and metabolic syndromes such as obesity, lipid metabolism disorders, insulin resistance, etc. It has become an important public health problem affecting women's physical and mental health.

However, increasing evidence illustrates a more multi-factorial and complex nature of the syndrome not previously apparent arising from a combination of genetic, psychological and environmental factors^3^. The relationship between gut microbiota dysbiosis and PCOS is attracted increasing attention. Kelley et al. found that the intestinal microbe composition changed significantly in a mice model study using letrozole to induce PCOS. The treatment of adolescent female mice with letrozole reduced the intestinal flora diversity and resulted in a species-specific and time-dependent shifts in the relative abundance of in particular, *Bacteroide*s and *Firmicutes*^4^. Another study showed *Lactobacillus*, *Ruminococcus*, and *Clostridium* abundance were lower in letrozole-treated PCOS rats^5^. Microbiota changes in mice and rat model were also demonstrated in human studies. Clinical trials showed a lower diversity and a modified phylogenetic profile in PCOS patient stool microbiomes^6^. In addition, the abundance of *Escherichia-Shigella* and *Streptococcus* in PCOS patients were increased, and the abundance of *Akkermansia* and *Rumenococcus* bacteria were decreased in other human studies^7^.

Metabolomics, using the qualitative and quantitative analysis of blood, urine, feces and other body fluids, improves our understanding of how specific metabolites, diseases and their phenotypic changes correlate. The human body is now considered as a superorganism as trillions of commensal microbes live on and inside the body, interacting through the process of metabolic exchange and "co-metabolism"^8^. The combination of serum metabolomics and 16S rRNA gene sequencing helps explain the close relationship between the gut microbiota and the host.

Using the non-targeted metabolomics technology to detect the serum and follicular fluid of PCOS patients, Xu et al. found that small molecules such as 1-methylhistidine, threonine and citric acid had underwent significant changes^9^. Zhao et al. found that discrete metabolites in PCOS patients are closely related to clinical symptoms^10^. Therefore, we recruited PCOS patients and healthy controls (HC) to study the relationship between the gut microbiota and metabolic changes in PCOS patients. Our research shows that the changes in the gut microbiota are related to host metabolism in PCOS patients, and the microbiota profile associated with the psychological state of PCOS, which provided a new perspective to explain the etiology and pathogenesis of PCOS.

## Results

### Baseline information

The study subjects were all women from the Pixian area of Chengdu, China. There was no significant difference in age between the PCOS patients and the healthy subjects (p > 0.05). The BMI of PCOS patients was increased compared with health subjects (p < 0.05), but the mean value is lower than 24. In a Chinese study, the authors recruited 999 volunteers with PCOS in Southern China, finding that the proportion of PCOS patients with a BMI above 23 kg/m^2^ was 34.63%^11^. It suggests that PCOS patients in China tend to have a lower BMI compared to world average, approximately 50% of PCOS women are overweight or obese^12^. Compared with healthy controls, the serum testosterone (T), luteinizing hormone (LH), LH/follicle-stimulating hormone (FSH) ratio and fasting insulin levels of PCOS patients were higher than those of the control group (p < 0.05). Further, the quality of life score SF-36 was lower than that of the control group (p < 0.01) (Table 1).

**Table 1:** Comparison of general information and serum hormone levels.

Items	PCOS (n = 20)	HC (n = 20)	*p*-value
Age (year)	28.95 ± 5.83	26.75 ± 5.46	0.226
BMI (kg/m^2^)	23.81 ± 2.02	22.54 ± 1.29	0.023
LH	12.63 ± 5.75	5.09 ± 1.65	0
FSH	4.32 ± 1.38	5.14 ± 1.58	0.09
LH/FSH	3.09 ± 1.58	1.01 ± 0.29	0
T	0.61 ± 0.37	0.24 ± 0.12	0
Insulin	12 ± 7.35	7.8 ± 2.71	0.025
SF-36	111.5 ± 13.27	123.8 ± 10.55	0.002

### Potential serum metabolomic biomarkers for PCOS

**Figure 1:**
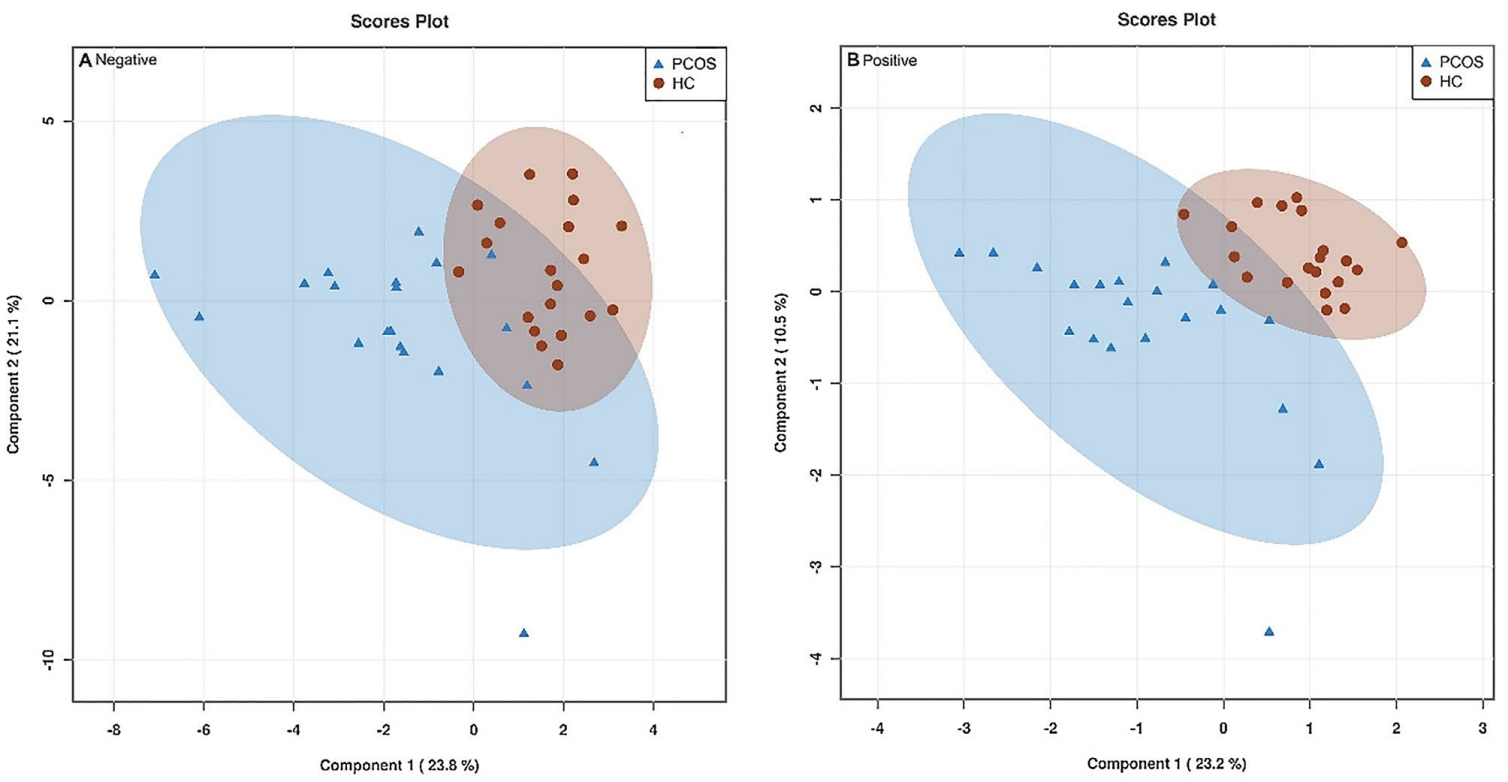
The PLS-DA plot of serum metabolomics in PCOS patients (blue triangles) and healthy controls (red circles). ESI(−), R2Y = 0.627, Q2 = 0.421 (**A**); ESI(+), R2Y = 0.761, Q2 = 0.580 (**B**). PCOS, n = 20, Healthy controls, n = 20.

We found 513 discernible features in positive ion mode, and 202 features in negative ion mode (supplementary Tables 1 and 2). In order to determine the difference in the metabolites between the two groups in our experiment design, partial least squares discriminant analysis (PLS-DA) was applied. The PLS-DA Scores plot is shown in Fig. 1, from which it can be seen that HC group and PCOS group are completely separated. The Variable Importance in the Projection (VIP) value of the PLS-DA model (threshold > 1) was adopted, combined with an independent sample T-test (P < 0.05) to search for different metabolites. A total of 15 different metabolites were detected (p < 0.05, VIP > 1, Table 2). The concentration of lysophosphatidylcholine (LPC) (18:2, 20:3, 18): 1, P-16:0, 17:0, 15:0, 18:3, 20:4), Phosphatidylcholine (PC), Ganglioside GA2 (d18:1/16: 0)), 1-Linoleoylglycerophosphocholine were higher in the PCOS group, while phosphoniodidous acid, bilirubin, nicotinate beta-d-ribonucleotide and citric acid concentrations were lower. Using MetaboAnalyst 3.0 to further analyze the differential metabolites and their related metabolic pathways, we found metabolites related to glycerophospholipid metabolism, such as LPC (18:2, 20:3, 18:1, P-16:0, 17:0, 15: 0,18:3,20:4), PC, 1-linoleoylglycerophosphocholine were enriched in the PCOS group; conversely, metabolites that related to energy metabolism, such as citric acid in the tricarboxylic acid cycle, the intermediate product nicotinate beta-d-ribonucleotide in the nicotinate and niacinamide metabolic pathway were significantly reduced in the PCOS group (Table 2).

**Table 2:** Identified serum metabolites and related metabolic pathways between PCOS and control.

Mode	ID	Metabolite	M/Z	P	FC	VIP value	Related pathway
					(PCOS/HC)		
ESI(−)	Var_164	LPC(18:2)	564.33	0	1.29	8.46	Glycerophospholipid metabolism
ESI(+)	Var_360	LPC(18:1)	522.36	0.008	1.3	6	Glycerophospholipid metabolism
ESI(−)	Var_200	1-Linoleoylglycerophosphocholine	1083.66	0	1.54	4.02	Glycerophospholipid metabolism
ESI(−)	Var_174	LPC(20:4)	588.33	0.005	1.33	3.31	Glycerophospholipid metabolism
ESI(−)	Var_30	Citric acid	191.02	0.035	0.55	2.56	TCA cycle
ESI(+)	Var_407	Bilirubin	585.27	0.01	0.54	1.77	Porphyrin metabolism
ESI(−)	Var_133	LPC(15:0)	480.31	0	1.23	1.68	Glycerophospholipid metabolism
ESI(+)	Var_511	Ganglioside GA2 (d18:1/16:0)	1087.67	0.002	2.4	1.51	Glycosphingolipid biosynthesis
ESI(+)	Var_112	Phosphoniodidous acid	198.88	0.026	0.23	1.38	–
ESI(−)	Var_163	LPC(18:3)	562.32	0.001	1.37	1.31	Glycerophospholipid metabolism
ESI(+)	Var_380	LPC(20:3)\PAF C-16	546.36	0.035	1.23	1.3	Glycerophospholipid metabolism
ESI(+)	Var_244	Nicotinate beta-d-ribonucleotide	337.06	0.014	0.19	1.18	Nicotinate and nicotinamide metabolism
ESI(−)	Var_158	PC(6:2/14:2)	556.32	0.001	1.25	1.17	Glycerophospholipid metabolism
ESI(+)	Var_346	LPC(17:0)	510.36	0.013	1.37	1.15	Glycerophospholipid metabolism
ESI(+)	Var_322	LPC(P-16:0)	480.33	0.026	1.19	1.06	Glycerophospholipid metabolism

M/Z, mass-to-charge ratio; ESI(−), negative ion scanning mode; ESI(+), positive ion scanning mode; VIP, variable important in projection; FC, fold change.

### The altered gut microbiota in women with PCOS

**Figure 2:**
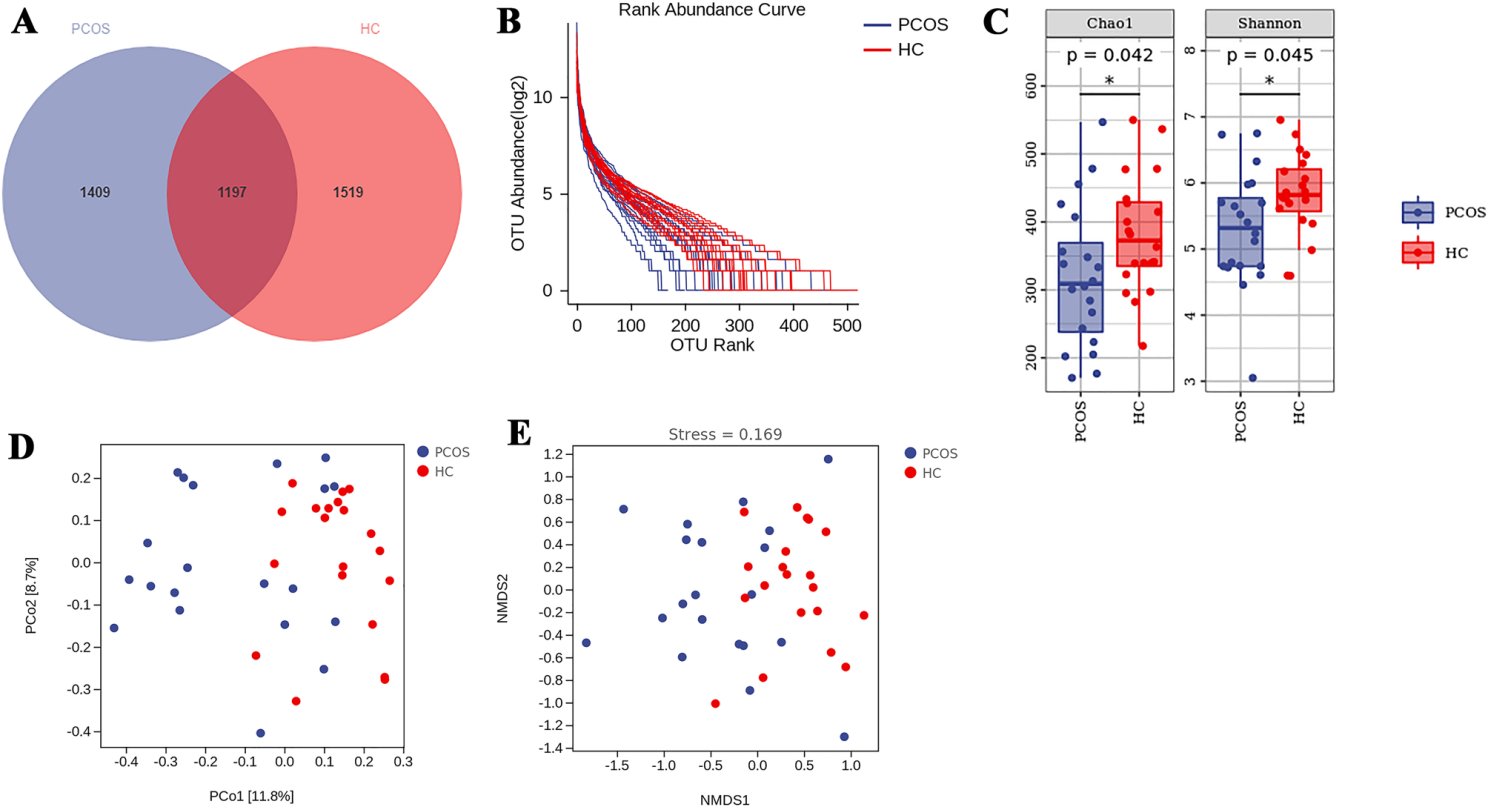
The dysbiosis of gut microbiota in the PCOS group. The ASVs Venn diagram of the PCOS and control (**A**); the rank abundance curve (**B**); alpha diversity, Chao1 index and Shannon index (**C**); PCoA analysis based on Bray–Curtis distance (**D**); NMDS analysis based on Bray–Curtis distance (stress = 0.169) (**E**).

We performed 16S rRNA gene V3-V4 regions sequencing to evaluate the gut microbiota of the PCOS patients. Using QIIME2 (2019,4), a total of 4125 ASVs were identified. The number of overlapped ASVs between the two groups was 1179, and the PCOS group had 1409 unique ASV sequences, while the healthy controls had 1519 unique ASV sequences (Fig. 2A). The rank abundance curve demonstrates the species richness and evenness. At ASV level, we found the rank-abundance curves of PCOS showed downward trends compared with the control group, suggesting the diversity of gut microbiota in the PCOS group was reduced (Fig. 2B). To further verify the difference of diversity and evenness between the two groups, we did alpha diversity analysis. It reveals the Chao1 index and Shannon index in the PCOS group were significantly lower than those of the healthy controls (P < 0.05) (Fig. 2C). Principal coordinates analysis (PCoA) and non-metric multidimensional scaling (NMDS) analysis of all the subjects displayed the dysbiosis caused by PCOS (Fig. 2D,E), in accordance with previous studies^13,14^.

**Figure 3:**
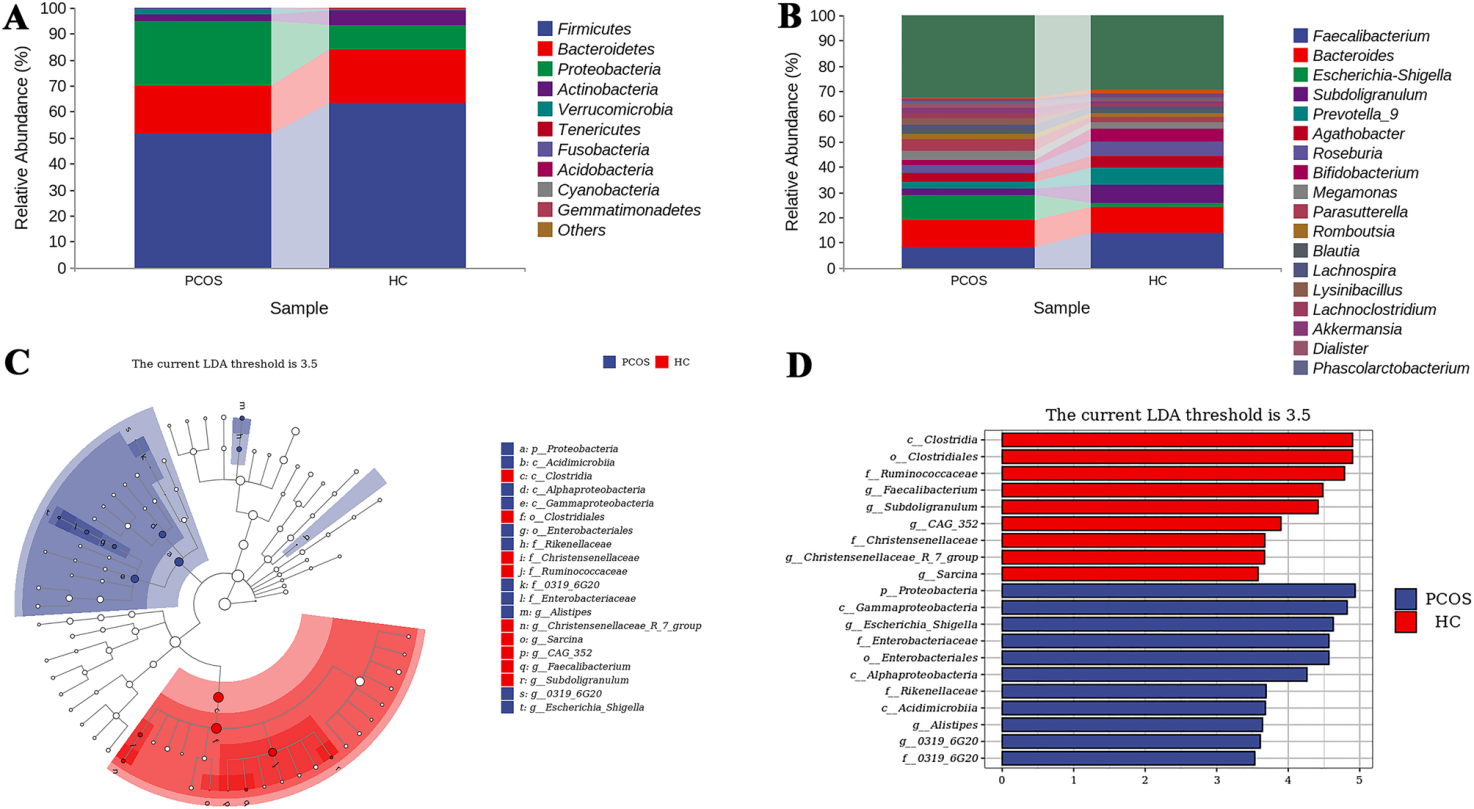
Gut microbiota composition differences between PCOS and HC. The relative abundance of the gut microbiome at phylum level (Top 10) (**A**); and at genus level (**B**); Lefse analysis taxonomy branch diagram (**C**); Lefse analysis LDA histogram provided key features of each group (LDA > 3.5) (**D**).

We then focused on the differences between groups on the taxonomic levels. At the phylum level, compared with healthy controls, the abundance of the *Firmicutes*, *Bacteroidetes*, *Actinobacteria*, *Tenericutes*, and *Gemmatimonadetes* in the PCOS group were decreased, and the proportion of *Proteobacteria*, *Verrucomicrobia*, *Fusobacteria*, *Acidobacteria*, and *Cyanobacteria* were increased (Fig. 3A supplementary Table 3). Among the top ten most abundant genera, the proportions of *Escherichia-Shigella*, *Megamonas* and *Parasutterella* in the PCOS group were increased, while the levels of the remaining seven genera were decreased (Fig. 3B supplementary Table 4. LefSe analysis showed that *Escherichia-Shigella, Alistipes* and an unnamed *genus 0319_6G20* belonging to *Proteobacteria* were the most important characteristic genera of the PCOS group (LDA > 3.5) (Fig. 3C,D).

### Correlation analysis of serum metabolites and intestinal microbiota in PCOS group

**Figure 4:**
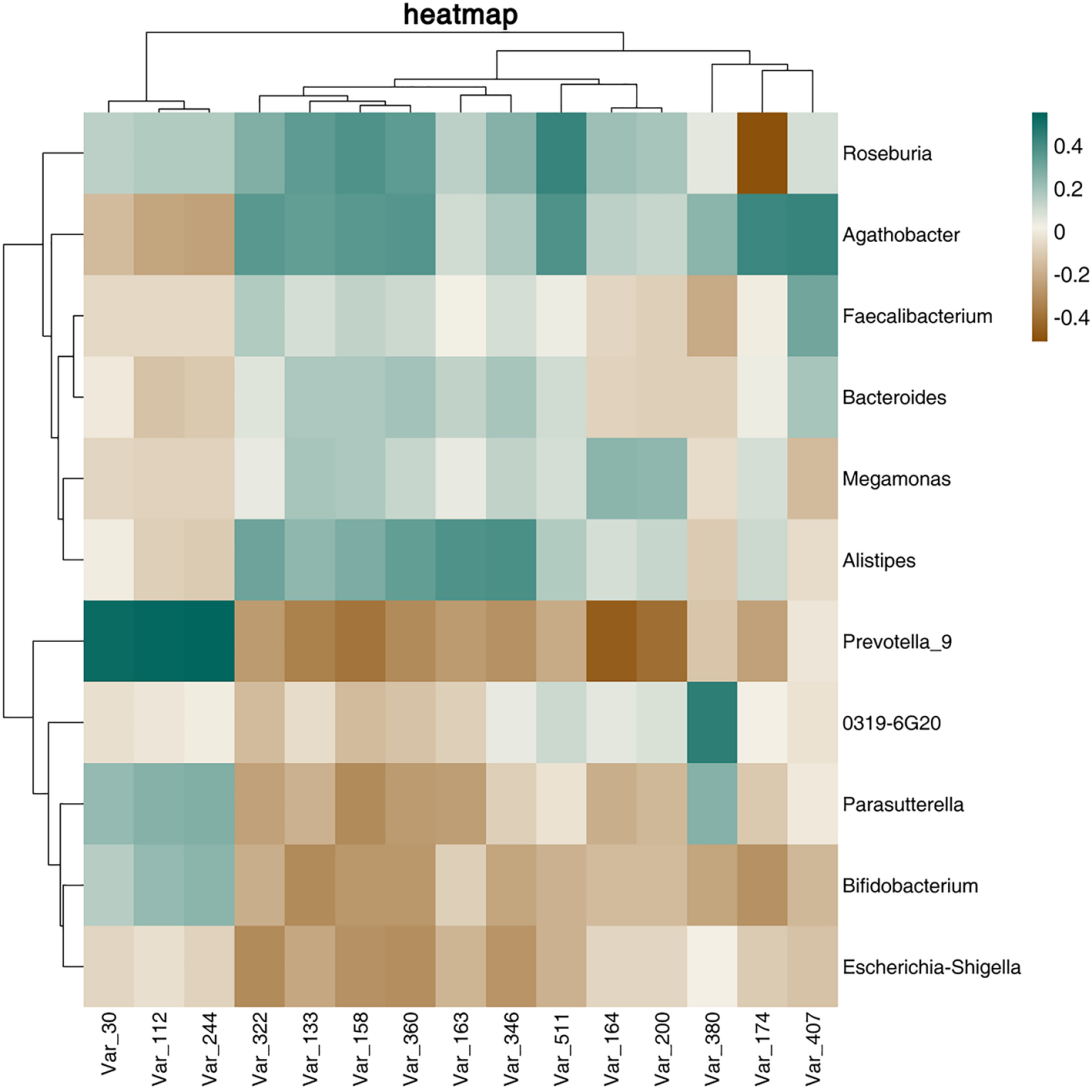
Heat map of Pearson correlation analysis between gut microbiota and serum metabolites in PCOS patients.

The Pearson correlation analysis between 15 important metabolites in serum and the gut microbiota of PCOS patients was carried out at the genus level. We found that *Prevotella_9* was positively correlated with the concentrations of Phosphoniodidous acid, nicotinate beta-d-ribonucleotide, and citric acid, and negatively correlated with the concentrations of LPC (18:2) and 1-Linoleoylglycerophosphocholine; *Roseburia* was negatively correlated with the concentrations of LPC (20:4), while the characteristic *genus 0319_6G20* of PCOS is positively correlated with the concentration of LPC (20:3) (Pearson Correlation Coefficient > 0.45) (Fig. 4).

## Discussion

In this study, we found the BMI of PCOS patients was increased compared with health subjects, but the mean is lower than 24. In a Chinese study, the authors recruited 999 volunteers with PCOS in Southern China, finding that the proportion of PCOS patients with a BMI above 23 kg/m^2^ was 34.63%^13^. It suggests that PCOS patients in China tend to have a lower BMI compared to world average, approximately 50% of PCOS women are overweight or obese^14^.

This study revealed a significant correlation between serum small molecular metabolites and the gut microbiota in PCOS patients, based on the analysis of the relative differences in serum metabolites in the PCOS and healthy controls. The important metabolic pathways were screened and 15 characteristic metabolites in serum were identified, which we suggest as potential biomarkers in future PCOS research. The 16S sequencing of fecal samples displayed a compositional dysbiosis of gut microbiota in the PCOS group. Furthermore, correlation analysis showed that the gut microbiota was associated with alterations in specific serum metabolites.

In this study, we found that in contrast to healthy controls, among the 15 different serum metabolites in PCOS samples, 13 are relating to the glycerophospholipid metabolism pathway, and 2 to energy metabolism, suggesting significant abnormalities with fat metabolism in patients with PCOS. Dysfunction of glycerophospholipid in PCOS patients' follicles play a discernible association with declining the 2 pronuclei (PN) fertilization rate during IVF procedure^15^. The abnormal PC/LPC ratio in PCOS, which may result in changes in serum arachidonic acid concentration, was regulated by circulating insulin and androgens. Further understanding of the molecular mechanisms that lead to the altered lipid profiles identified here, together with genomic and proteomic studies, may provide new insights into the pathogenic mechanisms of PCOS and inform novel therapeutic strategies^16^.

Following analysis of the alpha and beta diversity of the gut microbiota of the PCOS and control group showed that there is an imbalance in the microbial composition of PCOS patients, which is consistent with previous studies^6,7,17^. Compared with the control group, the ratio of *Firmicutes/Bacteroidetes* in PCOS decreased, while the *Proteobacteria, Verrucomicrobia* and *Fusobacteria* increased, and the *Actinobacteria* decreased. Generally, a healthy human gut microbial community is mainly composed of *Firmicutes* and *Bacteroidetes*, and the decline in the ratio of *Firmicutes*/*Bacteroidetes* is implicated in obesity^18^. The abundance of *Proteobacteria* and *Verrucomicrobia* in the gut of patients with type 2 diabetes is significantly increased^19^. In the intestinal microbial community, *Actinomycetes* account for a relatively small proportion, comparing to *Firmicutes* and *Bacteroidetes,* but studies have found that it plays an important role in maintaining the homeostasis of the intestinal microbiota. The genus of *Actinomycota* can produce large amounts of short-chain fatty acids (SCFAs), which provide energy for intestinal epithelial cells and protect the intestinal barrier from bacterial infections^20^. *Fusobacterium* is a commensal organism common in the oral cavity and known to be pathogenic under some conditions, associated with several human diseases, especially gastrointestinal disorders^21^.

Further, findings in this study show that among the top ten genera in relative abundance, the proportion of *Escherichia-Shigella*, *Megamonas*, and *Lachnospira* in the PCOS group were increased, while in the healthy control group and beneficial *Roseburia* and *Bifidobacterium* are increased *Escherichia-Shigella*, *Alistipes* and an unnamed genus *0319_6G20* belonging to *Proteobacteria* were identified as the most important characteristic genus in the PCOS group. Studies have shown that *Escherichia-Shigella*^22^ and *Alistipes*^23^ are significantly increased in the gut microbiota of patients with depression. In our study, based on the SF-36 questionnaire survey, we also found that the quality of life scores of the PCOS patients were significantly lower than those of healthy controls, indicating a correlation between depression and dysbiosis of gut microbiota in this population^24^. The brain-gut-axis is an interrelated system that affects both neural functions and eating behaviour^25^. Changes in gut microbiota affect the brain's physiological, behavioral, and cognitive functions through the influence of hormones, immune factors, and metabolites^26^ *Alistipes* is an indole-positive organism, and therefore decreases serotonin availability, which is associated with depression^23^. A previous study has displayed that *Escherichia/Shigella* is negatively correlated with the concentration of ghrelin^7^. Serotonin, peptide YY (PYY) and ghrelin are mediators of the brain–gut axis. This may shed new light on why PCOS patients are more prone to depression than healthy controls. Nevertheless, further mechanism studies are needed to prove whether the changes of gut microbiota are associated with the depression tendency.

Correlation analysis shows that some key gut microbial members were associated with the potential serum biomarkers of PCOS. Findings illustrate that among the top ten abundant bacterial genera all subjects in this study, the abundance of *Prevotella* was significantly reduced in PCOS patients, and negatively correlated with the serum metabolites LPC(18:2) and 1-Linoleoylglycerophosphocholine, and positively correlated with the concentration of β-nicotinic acid nucleotide and citric acid; *Roseburia* was negatively correlated with the concentration of LPC(20:4), while the *0319_6G20* genus and LPC(20:4) were positively correlated (cor > 0.45).

LPCs are essential substances and they are associated with metabolic disorders, such as inflammatory diseases^27^. In-vivo studies showed LPC production by hydrolysis of phosphatidylcholine induced by phospholipase A2, the main phospholipid component of oxidized low-density lipoprotein, is related to the occurrence of atherosclerosis. LPC can activate RhoA, a GTPase protein, through the PKC-α pathway and thus cause the dysfunction of the endothelial barrier. It also damage thw endothelial cells by preventing the synthesis of endogenous relaxing factors, as well as increasing the expressions of monocyte chemotactic protein-1 and interleukin-8 in endothelial cells through the activation of the NADH/NADPH oxidase system, which can lead to atherosclerosis and inflammatory diseases^28,29^. Some studies showed that LPCs can cause insulin resistance in diabetic patients but meanwhile lower the blood sugar^30^. Knowledge of this apparently opposing effect of LPC on human health is currently limited, and is an area for further research. In this study the LPCs of PCOS patients were significantly increased, supporting a plausible reason for the relatively high morbidity rate and cardiovascular disease in PCOS patients.

1-Linoleoylglycerophosphocholine, the product of PC metabolic pathway, was previously associated with heightened insulin resistance^31^. Our clinical data showed that PCOS group fasting insulin levels are within the normal range, but higher than the HC group, suggesting 1-Linoleoylglycerophosphocholine concentration can predict the rise of insulin resistance. Several studies have shown that a range of fatty acids represented by LPC appear at elevated levels in various diseases and are closely associated with gut microbiota^32,33^. *Prevotella* is considered as a gut microbial commensal in healthy human who consumes plant-rich diet, thus it is cosidered as a beneficial microbe^34^. However, *Prevotella* has also been shown to be associated with various diseases such as hypertension^33^, rheumatoid arthritis, periodontitis, and metabolic disorders^35^. Interestingly, in our study, *Prevotella* is associated with bile acid levels, including concentration of chenodeoxycholic acid (CDCA) and ursodeoxycholic acid (UDCA) which were positively correlated. Bile salts possess direct antimicrobial activities, thus being able to shape the structure of gut microbiota^36^. On the other side, gut microbes are able to produce bile salt hydrolases to deconjugate bile acids^37^. Bile acids can act as signaling molecules that regulate host metabolism by binding to the nuclear receptor farnesoid X receptor (FXR) and the Takeda G-protein coupled bile acid receptor TGR5, involving in the regulation of lipid metabolism^38^. The cause might be *Prevotella* is a large genus that includes more than 50 different species. The limitations of the resolution *Prevotella* genera which does not take into account species and strain level attributes which most likely account for the good and bad effects.

The concentration of nicotinate beta-d-ribonucleotide and citric acid in PCOS patients were lower than those in healthy controls. Nicotinate beta-d-ribonucleotid is a precursor for the synthesis of nicotinamide adenine dinucleotide (NAD+), which is essential for cell energy metabolism, cell protection and biosynthesis processes^39^. Studies have shown that NAD+ is negatively correlated with PCOS: The concentration of NAD+ in ovarian granulosa cells (GCs) in PCOS patients is significantly lower than that in healthy controls. Restoring NAD+ levels in PCOS patients can reduce the mitochondrial dysfunction of GCs^40^. Citric acid is an important intermediate product of the tricarboxylic acid cycle, which can reduce lipid peroxidation and reduce inflammation^41^. In a PCOS mouse model, the kidney tricarboxylic acid cycle products (citric acid, fumaric acid and succinic acid) and NAD+ levels were significantly altered^42^. There are consistent with the results of this study suggesting that oxidative stress and energy metabolism disorders in PCOS patients, which could arise through one of the the pathogenesis indicators of PCOS. Previous studies also found that after 6 weeks of ingesting whole grains and high dietary fiber diets, healthy, overweight adults with high abundance of *Prevotella* lost more body weight than those with low *Prevotella* abundance^43^. This may relate to its effective utilization of complex carbohydrates^44^, improving glucose metabolism, and therefore promoting glycogen storage^45^. The Pearson correlation analysis showed *Prevotella* was positively correlated with of citric acid and β-nicotinic acid nucleotides concentration, which is related to energy metabolism, suggesting that *Prevotella* may be related to energy metabolism and thus influence the metabolism of PCOS patients.

*Roseburia* is a genus of bacteria that produce acetate, propionate and butyrate. Its abundance in the gut of people with metabolic and inflammatory diseases is reduced^46^, and its abundance is inversely correlated to the occurrence of coronary atherosclerosis^47^. *Roseburia* ferments plant fibers and produces organic acids, one of which is butyrate, which can improve colon movement, immune maintenance and anti-inflammatory effects^48^. This study found that *Roseburia* was negatively correlated with the concentration of LPC (20:4). Other studies have also found the elevated concentration of butyrate-producing bacteria, with reduced levels of certain plasma LPC^49^. Butyric acid, a short-chain fatty acid (SCFAs), acts as a signaling molecule, notably through the G-protein coupled receptors GPR43/FFAR2 and GPR41/FFAR3. Activation of GPR43 on L-cells increases secretion of glucagon-like peptide-1 (GLP-1) and acetate induces anti-lipolytic activity and improves glucose and lipid metabolism^50^.

The findings are presented here within the limitations of the study: the sample size is relatively small, stated correlations have not yet been further verified, and our research is based on 16S rRNA sequencing results within the limits of resolution. A future study is planned to compare these finding in higher sample sizes and in animal models, which should further extend our understanding through functional analysis of the microbiota. The longer-term aim of this work is to design metatranscriptomic and metametabolomic analysis techniques and possibly selectively managing gut microbiota of PCOS patients. In this study, for the first time, the results of serum metabolomics and gut microbiota were combined to explore the possible mechanism of metabolic disorders in PCOS patients. The findings support and inform a biological treatment of PCOS in patients. In summary, non-targeted metabolomics and 16S rRNA gene sequencing revealed characteristic changes in fecal metabolites and gut microbiota of PCOS patients. *Escherichia-Shigella, Alistipes,* and an unnamed genus *0319_6G20* in the phylum *Proteobacteria* were observed in PCOS group patients. Linoleoylglycerophosphocholine, LPC (18:2, 20:3, 18:1, P-16:0, 17:0, 15:0, 18:3, 20:4), phosphoniodidous acid, PC, bilirubin, ganglioside GA2, β-nicotinic acid nucleotide, citric acid are characteristic metabolites in the PCOS group. In these patients, there is a close correlation between the gut microbiota and the serum metabolites. Study of the characteristic gut microbiota and its metabolites should be further extended to, for example, examine relationships to the physiological and emotional changes in patients. In future research, we plan to use the findings here to design a target strain transplantation and when complete, compare with the findings of this study to develop a biogenic and effective treatment for PCOS.

## Methods

### Participants

From November 2015 to December 2017, PCOS patients (PCOS group, n = 20) attending the outpatient clinic of Pixian Hospital of Traditional Chinese Medicine, Republic of China were recruited. The gynecologists used Rotterdam criteria to diagnose PCOS. Participants who presented with two out of three following criteria were diagnosed as PCOS: (1) clinical and/or biochemical hyperandrogenism (hirsutism and/or an increased testosterone concentration); (2) oligo- and/or anovulation (menstrual cycles > 35 days and/or the absence of menstruation for at least 3 months); (3) polycystic ovaries (assessed through gynecological ultrasound or medical history).

The healthy control group aged 18–40 years old (HC group, n = 20) had no history of diagnosed PCOS and did not meet the Rotterdam criteria. All participants met the exclusion criteria, including no use of oral contraceptives, antiandrogens, or insulin sensitizers in the past three months; pregnancy; other known disorders that can cause hyperandrogen and ovulation, such as 21-hydroxylase deficiency, congenital adrenal hyperplasia, Cushing’s syndrome, androgen secretory tumors, thyroid disease and hyperprolactinemia; any mental or organic disease; the use of corticosteroids or sex steroids; drug and alcohol abuse in the past 2 years; and the use of antibiotics, probiotics or prebiotics in the past 3 months. All subjects were from Pidu district Chengdu city region in Sichuan province, China, to minimize any confounding influences related to geographical location. This study was approved by the Ethics Committee of the Hospital of Traditional Chinese Medicine in Pixian Chengdu, China (2015KL-001). Every subject completed an informed consent form in accordance with the Declaration of Helsinki.

### Collection of peripheral venous blood and stool samples

All subjects’ peripheral venous blood was collected on the third day of the menstrual cycle and the levels of sex hormones (luteinizing hormone (LH), follicle stimulating hormone (FSH), and testosterone (T)) were determined to ensure that no abnormalities were present. Stool samples were obtained from the participants 3–5 days after menstruation. Sterile plastic spoons and plastic tubes were used by each subject to collect about 10 g of fresh stool samples. The blood and fecal samples were transported within 2 h and preserved at − 80 °C until further processing.

### Non-targeted metabolomics analysis

The serum samples were thawed from − 80 °C to room temperature in the dark. 300 μl of methanol solution (Dannstadt, Gennany) containing internal standard (5ug/mL, l-2-chloro-phenylalanine) was added into 100ul serum, vortexed for 1 min, and centrifuged at 13,000 rpm for 15 min at 4 °C. 200 μl of the supernatant was added into a sample vial for subsequent metabolomics analysis. In addition, an equal volume of each sample solution without blood to be tested was mixed together, serving as the QC (Quality Control).

The LC–MS detection platform was: liquid chromatography Agilent 1290 Infinity UHPLC, mass spectrometry Agilent 6538 UHD and Accurate-Mass Q-TOF /MS, chromatographic column XSelect HSS T3 (2.1 × 100 mm, 2.5 m). The mass spectrum data was collected by Accurate-Mass Q-TOF/MS, and the working mode divided into positive ion and negative ion mode. The gas temperature was 350 °C, the intake air volume 11 L/min, the fragmentation voltage 120 V, and the center of mass data collected in the range of 50–1000 m/z. Agilent Masshunter Qualitative Analysis B.04.00 software (Agilent Technologies, USA) was used to convert the raw data into a common (mz.data) format. The XCMS package^51^ in R was used to perform downstream analysis, such as peak identification, retention time correction, and automatic integration. We did internal standard normalization, and the number of sample features was screened according to the 80% principle. After that, we obtained a visualization matrix containing sample name, m/z-RT pair, and peak area. Importation of the visualization matrix into the R language platform was done, using the Pareto Scaling method for normalization, and to perform multivariate statistical analyses.

Access to cited metabolomics data: MTBLS3975 (http://www.ebi.ac.uk/metabolights/MTBLS3975)^52^.

### 16S rRNA gene sequencing

Stool samples were thawed in 4 °C water, centrifuged for 10 min, and DNA was extracted using Axygen Axy Prep DNA Gel Kit (Axygen, USA). The amplification method was applied, with primers and F:AYTGGGYDTAAAGNG R:TACNVGGGTATCTAATCC. Library prep for V3-V4 region, and the library pool was submitted to the Illumina MiSeq platform with 2 × 250 paired-end sequencing. Data were analyzed using QIIME2 (2019.4)^53^ software. Taxonomic results were summarized in supplementary Table 5. Accession to cite for these SRA data: PRJNA779930.

### Statistical analysis

Statistical analyses were performed using SPSS 22.0 version software (IBM Inc., USA). Continuous variables are described by mean and standard deviation (SD). If the changes in α and β diversity indexes of metabolites and microbial communities were normally distributed, a covariance analysis was performed; otherwise the non-parametric Kruskal Wallis test was used. Differences in metabolites and gut microbiota between the two groups were analyzed using two independent samples t test or Wilcoxon rank sum test. Partial least squares discriminant analysis (PLS-DA) was used to analyze the two groups of samples. VIP (Variable Importance in the Projection) values of PLS-DA model (threshold > 1) were used to search for differential metabolites combined with independent sample T-test (P < 0.05). The Pearson correlation was used to analyze the correlation between each discriminated metabolite and the gut microbiota. A two-tailed p < 0.05 threshold indicated that the differences were statistically significant.

## Supplementary Information

Supplementary Information 1.

Supplementary Information 2.

Supplementary Information 3.

Supplementary Information 4.

Supplementary Information 5.

Supplementary Information 6.

Supplementary Information 7.

Supplementary Table 1.

Supplementary Table 2.

Supplementary Table 3.

Supplementary Table 4.

Supplementary Table 5.

## Data Availability

The 16S rRNA gene sequencing datasets generated and analyzed during the current study are available in the NCBI (https://www.ncbi.nlm.nih.gov/bioproject/PRJNA779930/), and Non-targeted metabolomics analysis datasets generated and analyzed during the current study are available in the Metbolights (http://www.ebi.ac.uk/metabolights/MTBLS3975).
