# Supplementary figures and images for: Gut microbiome in PCOS associates to serum metabolomics: a cross-sectional study
Source: Sci Rep. 2022 Dec 23;12:22184. doi: 10.1038/s41598-022-25041-4 (PMC9789036; doi:10.1038/s41598-022-25041-4)

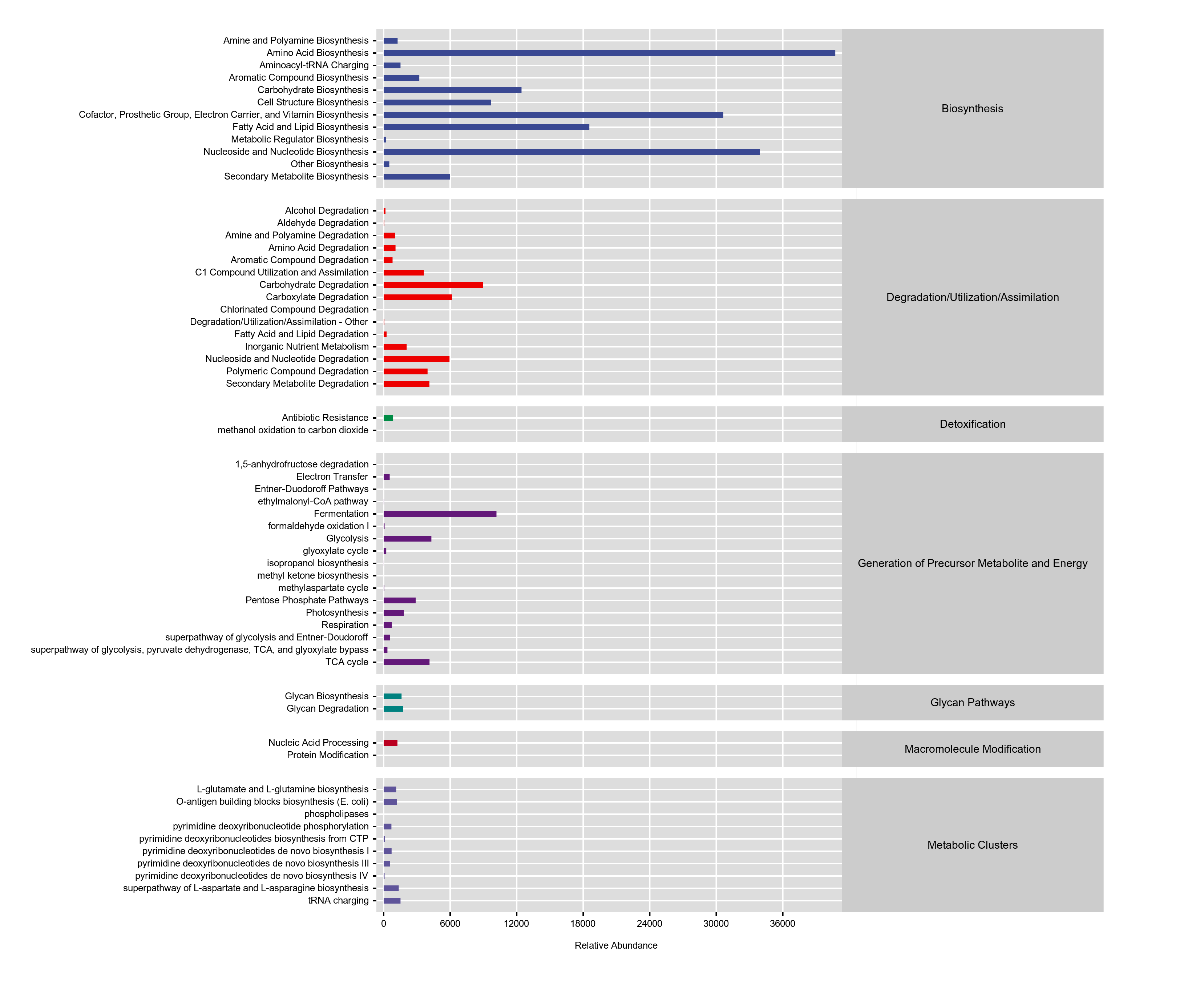

Supplement: Supplementary file 3 — Supplementary Information 3. [file 41598_2022_25041_MOESM3_ESM.png]
