# Supplementary material for: Gut microbiome in PCOS associates to serum metabolomics: a cross-sectional study
Source: Sci Rep. 2022 Dec 23;12:22184. doi: 10.1038/s41598-022-25041-4 (PMC9789036; doi:10.1038/s41598-022-25041-4)
Supplement: Supplementary file 4 — Supplementary Information 4. [file 41598_2022_25041_MOESM4_ESM.docx]

| pathway | description | logFC | SE | Pvalues | adjPvalues |
| --- | --- | --- | --- | --- | --- |
| PWY0-1338 | polymyxin resistance | 1.314 | 1.451 | 0.3653 | 0.8503 |
| HCAMHPDEG-PWY | 3-phenylpropanoate and 3-(3-hydroxyphenyl)propanoate degradation to 2-oxopent-4-enoate | 1.267 | 1.526 | 0.4063 | 0.8503 |
| PWY-6690 | cinnamate and 3-hydroxycinnamate degradation to 2-oxopent-4-enoate | 1.267 | 1.526 | 0.4063 | 0.8503 |
| PWY-6944 | androstenedione degradation | 1.217 | 0.8453 | 0.15 | 0.8503 |
| ARGDEG-PWY | superpathway of L-arginine, putrescine, and 4-aminobutanoate degradation | 1.207 | 1.39 | 0.3853 | 0.8503 |
| ORNARGDEG-PWY | superpathway of L-arginine and L-ornithine degradation | 1.207 | 1.39 | 0.3853 | 0.8503 |
| PWY0-1277 | 3-phenylpropanoate and 3-(3-hydroxyphenyl)propanoate degradation | 1.182 | 1.496 | 0.4295 | 0.8503 |
| AST-PWY | L-arginine degradation II (AST pathway) | 1.12 | 1.469 | 0.4459 | 0.8503 |
| AEROBACTINSYN-PWY | aerobactin biosynthesis | 1.08 | 1.179 | 0.3597 | 0.8503 |
| PWY-6629 | superpathway of L-tryptophan biosynthesis | 1.079 | 1.292 | 0.4037 | 0.8503 |
| ECASYN-PWY | enterobacterial common antigen biosynthesis | 1.075 | 1.293 | 0.4059 | 0.8503 |
| ORNDEG-PWY | superpathway of ornithine degradation | 1.073 | 1.366 | 0.4321 | 0.8503 |
| PWY0-42 | 2-methylcitrate cycle I | 1.053 | 1.467 | 0.473 | 0.8503 |
| METHGLYUT-PWY | superpathway of methylglyoxal degradation | 1.051 | 1.277 | 0.4103 | 0.8503 |
| PWY-5183 | superpathway of aerobic toluene degradation | 1.02 | 0.6313 | 0.1063 | 0.8503 |
| GLYOXYLATE-BYPASS | glyoxylate cycle | 1.019 | 1.428 | 0.4753 | 0.8503 |
| GLYCOL-GLYOXDEG-PWY | superpathway of glycol metabolism and degradation | 1.012 | 1.145 | 0.3769 | 0.8503 |
| PWY-7295 | L-arabinose degradation IV | 1.001 | 0.684 | 0.1435 | 0.8503 |
| PWY-5415 | catechol degradation I (meta-cleavage pathway) | 0.9644 | 1.593 | 0.545 | 0.8503 |
| TYRFUMCAT-PWY | L-tyrosine degradation I | 0.9608 | 1.893 | 0.6118 | 0.8719 |
| PWY-1622 | formaldehyde assimilation I (serine pathway) | 0.9566 | 0.6829 | 0.1613 | 0.8503 |
| TCA-GLYOX-BYPASS | superpathway of glyoxylate bypass and TCA | 0.9556 | 1.413 | 0.4988 | 0.8503 |
| PWY-6713 | L-rhamnose degradation II | 0.9035 | 0.5956 | 0.1293 | 0.8503 |
| P341-PWY | glycolysis V (Pyrococcus) | 0.8862 | 1.073 | 0.4089 | 0.8503 |
| PWY-7094 | fatty acid salvage | 0.8811 | 1.634 | 0.5897 | 0.859 |
| PWY-7431 | aromatic biogenic amine degradation (bacteria) | 0.8802 | 2.016 | 0.6623 | 0.9052 |
| PWY-6728 | methylaspartate cycle | 0.8765 | 0.7258 | 0.2272 | 0.8503 |
| PPGPPMET-PWY | ppGpp biosynthesis | 0.8687 | 1.152 | 0.4509 | 0.8503 |
| PWY-5741 | ethylmalonyl-CoA pathway | 0.8667 | 0.8645 | 0.3161 | 0.8503 |
| UBISYN-PWY | superpathway of ubiquinol-8 biosynthesis (prokaryotic) | 0.8293 | 1.402 | 0.5541 | 0.8503 |
| PWY-5180 | toluene degradation I (aerobic) (via o-cresol) | 0.8235 | 1.292 | 0.5238 | 0.8503 |
| PWY-5182 | toluene degradation II (aerobic) (via 4-methylcatechol) | 0.8235 | 1.292 | 0.5238 | 0.8503 |
| PWY-5855 | ubiquinol-7 biosynthesis (prokaryotic) | 0.8149 | 1.424 | 0.5672 | 0.8503 |
| PWY-5856 | ubiquinol-9 biosynthesis (prokaryotic) | 0.8149 | 1.424 | 0.5672 | 0.8503 |
| PWY-5857 | ubiquinol-10 biosynthesis (prokaryotic) | 0.8149 | 1.424 | 0.5672 | 0.8503 |
| PWY-6708 | ubiquinol-8 biosynthesis (prokaryotic) | 0.8149 | 1.424 | 0.5672 | 0.8503 |
| PWY-5747 | 2-methylcitrate cycle II | 0.8139 | 1.632 | 0.618 | 0.8719 |
| FAO-PWY | fatty acid &beta;-oxidation I | 0.8109 | 1.222 | 0.5068 | 0.8503 |
| ENTBACSYN-PWY | enterobactin biosynthesis | 0.7898 | 1.228 | 0.5201 | 0.8503 |
| P101-PWY | ectoine biosynthesis | 0.7857 | 0.7887 | 0.3192 | 0.8503 |
| GLYCOLYSIS-TCA-GLYOX-BYPASS | superpathway of glycolysis, pyruvate dehydrogenase, TCA, and glyoxylate bypass | 0.784 | 1.297 | 0.5455 | 0.8503 |
| PWY-3781 | aerobic respiration I (cytochrome c) | 0.7837 | 2.057 | 0.7032 | 0.9145 |
| P621-PWY | nylon-6 oligomer degradation | 0.7801 | 0.76 | 0.3046 | 0.8503 |
| PWY-5920 | superpathway of heme biosynthesis from glycine | 0.7554 | 1.138 | 0.5068 | 0.8503 |
| PWY-5420 | catechol degradation II (meta-cleavage pathway) | 0.7469 | 0.6002 | 0.2134 | 0.8503 |
| PWY-5430 | meta cleavage pathway of aromatic compounds | 0.7226 | 0.5854 | 0.2171 | 0.8503 |
| PWY-5419 | catechol degradation to 2-oxopent-4-enoate II | 0.7195 | 0.6197 | 0.2456 | 0.8503 |
| PWY-5178 | toluene degradation IV (aerobic) (via catechol) | 0.7166 | 0.4764 | 0.1326 | 0.8503 |
| PWY-3661 | glycine betaine degradation I | 0.7137 | 0.7331 | 0.3302 | 0.8503 |
| GLUCARDEG-PWY | D-glucarate degradation I | 0.7112 | 0.8041 | 0.3765 | 0.8503 |
| PWY-1361 | benzoyl-CoA degradation I (aerobic) | 0.7099 | 0.7231 | 0.3263 | 0.8503 |
| PWY-5647 | 2-nitrobenzoate degradation I | 0.7093 | 0.6228 | 0.2547 | 0.8503 |
| PWY-1541 | superpathway of taurine degradation | 0.7093 | 0.6533 | 0.2776 | 0.8503 |
| PWY-5654 | 2-amino-3-carboxymuconate semialdehyde degradation to 2-oxopentenoate | 0.7077 | 0.6269 | 0.259 | 0.8503 |
| GALACTARDEG-PWY | D-galactarate degradation I | 0.6936 | 1.203 | 0.5644 | 0.8503 |
| GLUCARGALACTSUPER-PWY | superpathway of D-glucarate and D-galactarate degradation | 0.6936 | 1.203 | 0.5644 | 0.8503 |
| THREOCAT-PWY | superpathway of L-threonine metabolism | 0.6877 | 0.5047 | 0.173 | 0.8503 |
| P105-PWY | TCA cycle IV (2-oxoglutarate decarboxylase) | 0.6844 | 1.214 | 0.5728 | 0.8503 |
| PWY-5651 | L-tryptophan degradation to 2-amino-3-carboxymuconate semialdehyde | 0.6689 | 0.5967 | 0.2623 | 0.8503 |
| PWY-5941 | glycogen degradation II (eukaryotic) | 0.6659 | 0.8906 | 0.4546 | 0.8503 |
| PWY0-321 | phenylacetate degradation I (aerobic) | 0.6653 | 0.6005 | 0.2679 | 0.8503 |
| PWY-6505 | L-tryptophan degradation XII (Geobacillus) | 0.665 | 0.5983 | 0.2664 | 0.8503 |
| PWY-6957 | mandelate degradation to acetyl-CoA | 0.6622 | 0.661 | 0.3164 | 0.8503 |
| NADSYN-PWY | NAD biosynthesis II (from tryptophan) | 0.6579 | 0.5912 | 0.2658 | 0.8503 |
| PWY-5181 | toluene degradation III (aerobic) (via p-cresol) | 0.6498 | 2.092 | 0.7562 | 0.9215 |
| PWY-181 | photorespiration | 0.6409 | 0.6375 | 0.3147 | 0.8503 |
| PWY-6071 | superpathway of phenylethylamine degradation | 0.6332 | 0.5975 | 0.2893 | 0.8503 |
| PWY-1501 | mandelate degradation I | 0.6271 | 0.6555 | 0.3387 | 0.8503 |
| ALL-CHORISMATE-PWY | superpathway of chorismate metabolism | 0.6266 | 1.066 | 0.5567 | 0.8503 |
| PWY-6107 | chlorosalicylate degradation | 0.6266 | 0.7919 | 0.4288 | 0.8503 |
| PWY-5823 | superpathway of CDP-glucose-derived O-antigen building blocks biosynthesis | 0.6265 | 0.3304 | 0.05794 | 0.8503 |
| LEU-DEG2-PWY | L-leucine degradation I | 0.6194 | 0.6715 | 0.3563 | 0.8503 |
| KDO-NAGLIPASYN-PWY | superpathway of (Kdo)2-lipid A biosynthesis | 0.6178 | 0.8752 | 0.4802 | 0.8503 |
| PWY-5860 | superpathway of demethylmenaquinol-6 biosynthesis I | 0.6131 | 0.9738 | 0.5289 | 0.8503 |
| PWY-5862 | superpathway of demethylmenaquinol-9 biosynthesis | 0.6131 | 0.9738 | 0.5289 | 0.8503 |
| PWY-1882 | superpathway of C1 compounds oxidation to CO2 | 0.6011 | 0.9974 | 0.5468 | 0.8503 |
| GALLATE-DEGRADATION-I-PWY | gallate degradation II | 0.5943 | 0.5562 | 0.2853 | 0.8503 |
| METHYLGALLATE-DEGRADATION-PWY | methylgallate degradation | 0.5939 | 0.5561 | 0.2855 | 0.8503 |
| PROTOCATECHUATE-ORTHO-CLEAVAGE-PWY | protocatechuate degradation II (ortho-cleavage pathway) | 0.5917 | 2.088 | 0.7769 | 0.9228 |
| 3-HYDROXYPHENYLACETATE-DEGRADATION-PWY | 4-hydroxyphenylacetate degradation | 0.5776 | 1.651 | 0.7264 | 0.9196 |
| KETOGLUCONMET-PWY | ketogluconate metabolism | 0.5769 | 0.4624 | 0.2122 | 0.8503 |
| PWY-6641 | superpathway of sulfolactate degradation | 0.5674 | 0.6441 | 0.3784 | 0.8503 |
| PWY-6185 | 4-methylcatechol degradation (ortho cleavage) | 0.567 | 1.948 | 0.771 | 0.9228 |
| PWY-5837 | 1,4-dihydroxy-2-naphthoate biosynthesis I | 0.5656 | 0.7696 | 0.4624 | 0.8503 |
| PWY-6165 | chorismate biosynthesis II (archaea) | 0.5628 | 0.6193 | 0.3635 | 0.8503 |
| DENITRIFICATION-PWY | nitrate reduction I (denitrification) | 0.562 | 0.739 | 0.447 | 0.8503 |
| PWY-5845 | superpathway of menaquinol-9 biosynthesis | 0.555 | 0.9134 | 0.5434 | 0.8503 |
| PWY-5850 | superpathway of menaquinol-6 biosynthesis I | 0.555 | 0.9134 | 0.5434 | 0.8503 |
| PWY-5896 | superpathway of menaquinol-10 biosynthesis | 0.555 | 0.9134 | 0.5434 | 0.8503 |
| P125-PWY | superpathway of (R,R)-butanediol biosynthesis | 0.5535 | 1.348 | 0.6813 | 0.9132 |
| VALDEG-PWY | L-valine degradation I | 0.553 | 0.6608 | 0.4027 | 0.8503 |
| PWY-5863 | superpathway of phylloquinol biosynthesis | 0.5485 | 0.7603 | 0.4706 | 0.8503 |
| PWY0-1533 | methylphosphonate degradation I | 0.5442 | 1.253 | 0.6642 | 0.9052 |
| PWY-6876 | isopropanol biosynthesis | 0.541 | 0.4645 | 0.2441 | 0.8503 |
| PWY-6182 | superpathway of salicylate degradation | 0.5387 | 2.011 | 0.7888 | 0.9228 |
| PWY-5028 | L-histidine degradation II | 0.5329 | 0.6546 | 0.4156 | 0.8503 |
| GALLATE-DEGRADATION-II-PWY | gallate degradation I | 0.5176 | 0.5752 | 0.3682 | 0.8503 |
| NAD-BIOSYNTHESIS-II | NAD salvage pathway II | 0.5001 | 0.8027 | 0.5333 | 0.8503 |
| PWY-5655 | L-tryptophan degradation IX | 0.4921 | 0.7118 | 0.4894 | 0.8503 |
| PWY-5417 | catechol degradation III (ortho-cleavage pathway) | 0.4651 | 2.008 | 0.8169 | 0.9228 |
| PWY-5431 | aromatic compounds degradation via &beta;-ketoadipate | 0.4651 | 2.008 | 0.8169 | 0.9228 |
| PWY-7098 | vanillin and vanillate degradation II | 0.4546 | 0.7072 | 0.5203 | 0.8503 |
| PWY-6338 | superpathway of vanillin and vanillate degradation | 0.454 | 0.7076 | 0.5211 | 0.8503 |
| PWY-7097 | vanillin and vanillate degradation I | 0.454 | 0.7076 | 0.5211 | 0.8503 |
| P281-PWY | 3-phenylpropanoate degradation | 0.4493 | 1.806 | 0.8035 | 0.9228 |
| PWY-7376 | cob(II)yrinate a,c-diamide biosynthesis II (late cobalt incorporation) | 0.44 | 0.7366 | 0.5503 | 0.8503 |
| CATECHOL-ORTHO-CLEAVAGE-PWY | catechol degradation to &beta;-ketoadipate | 0.4382 | 1.995 | 0.8262 | 0.9259 |
| PWY-6210 | 2-aminophenol degradation | 0.4355 | 0.7674 | 0.5704 | 0.8503 |
| HEMESYN2-PWY | heme biosynthesis II (anaerobic) | 0.423 | 0.7125 | 0.5527 | 0.8503 |
| GLUCOSE1PMETAB-PWY | glucose and glucose-1-phosphate degradation | 0.4126 | 0.9061 | 0.6488 | 0.8921 |
| P184-PWY | protocatechuate degradation I (meta-cleavage pathway) | 0.4036 | 0.6941 | 0.5609 | 0.8503 |
| HEME-BIOSYNTHESIS-II | heme biosynthesis I (aerobic) | 0.4015 | 1.179 | 0.7334 | 0.9196 |
| PWY-5345 | superpathway of L-methionine biosynthesis (by sulfhydrylation) | 0.376 | 0.7617 | 0.6216 | 0.8719 |
| PWY-5861 | superpathway of demethylmenaquinol-8 biosynthesis | 0.3746 | 0.6326 | 0.5537 | 0.8503 |
| PWY-7446 | sulfoglycolysis | 0.3705 | 0.5762 | 0.5202 | 0.8503 |
| REDCITCYC | TCA cycle VIII (helicobacter) | 0.3399 | 1.133 | 0.7641 | 0.9228 |
| PWY-5897 | superpathway of menaquinol-11 biosynthesis | 0.3338 | 0.591 | 0.5722 | 0.8503 |
| PWY-5898 | superpathway of menaquinol-12 biosynthesis | 0.3338 | 0.591 | 0.5722 | 0.8503 |
| PWY-5899 | superpathway of menaquinol-13 biosynthesis | 0.3338 | 0.591 | 0.5722 | 0.8503 |
| PWY-7315 | dTDP-N-acetylthomosamine biosynthesis | 0.3263 | 0.4454 | 0.4639 | 0.8503 |
| PYRIDOXSYN-PWY | pyridoxal 5'-phosphate biosynthesis I | 0.3161 | 0.8354 | 0.7051 | 0.9145 |
| PWY-5838 | superpathway of menaquinol-8 biosynthesis I | 0.3067 | 0.5698 | 0.5904 | 0.859 |
| PWY-5840 | superpathway of menaquinol-7 biosynthesis | 0.3007 | 0.5732 | 0.5998 | 0.8671 |
| PWY-4984 | urea cycle | 0.2899 | 1.081 | 0.7886 | 0.9228 |
| PWY-7347 | sucrose biosynthesis III | 0.2859 | 0.7713 | 0.7109 | 0.9145 |
| PWY0-845 | superpathway of pyridoxal 5'-phosphate biosynthesis and salvage | 0.2846 | 0.8021 | 0.7227 | 0.9196 |
| SUCSYN-PWY | sucrose biosynthesis I (from photosynthesis) | 0.2836 | 0.7654 | 0.711 | 0.9145 |
| PWY-5529 | superpathway of bacteriochlorophyll a biosynthesis | 0.2763 | 0.7073 | 0.696 | 0.9145 |
| PWY-7616 | methanol oxidation to carbon dioxide | 0.2711 | 0.7174 | 0.7055 | 0.9145 |
| PWY0-1415 | superpathway of heme biosynthesis from uroporphyrinogen-III | 0.258 | 1.046 | 0.8052 | 0.9228 |
| P381-PWY | adenosylcobalamin biosynthesis II (late cobalt incorporation) | 0.248 | 0.657 | 0.7058 | 0.9145 |
| PWY-5531 | chlorophyllide a biosynthesis II (anaerobic) | 0.239 | 0.6915 | 0.7296 | 0.9196 |
| PWY-7159 | chlorophyllide a biosynthesis III (aerobic, light independent) | 0.239 | 0.6915 | 0.7296 | 0.9196 |
| CHLOROPHYLL-SYN | chlorophyllide a biosynthesis I (aerobic, light-dependent) | 0.2263 | 0.6599 | 0.7317 | 0.9196 |
| PWY-5177 | glutaryl-CoA degradation | 0.2164 | 1.143 | 0.8498 | 0.9275 |
| CENTFERM-PWY | pyruvate fermentation to butanoate | 0.2154 | 0.9598 | 0.8225 | 0.9242 |
| PWY-6396 | superpathway of 2,3-butanediol biosynthesis | 0.2119 | 1.044 | 0.8391 | 0.9275 |
| PWY-5918 | superpathay of heme biosynthesis from glutamate | 0.2114 | 1.04 | 0.8389 | 0.9275 |
| PWY1G-0 | mycothiol biosynthesis | 0.2033 | 0.718 | 0.777 | 0.9228 |
| PWY-6590 | superpathway of Clostridium acetobutylicum acidogenic fermentation | 0.1857 | 0.9413 | 0.8437 | 0.9275 |
| PWY0-1241 | ADP-L-glycero-&beta;-D-manno-heptose biosynthesis | 0.1802 | 0.5439 | 0.7404 | 0.92 |
| PWY-7373 | superpathway of demethylmenaquinol-6 biosynthesis II | 0.1709 | 0.1163 | 0.1417 | 0.8503 |
| PWY0-1479 | tRNA processing | 0.1664 | 0.8054 | 0.8363 | 0.9275 |
| PWY-7003 | glycerol degradation to butanol | 0.1661 | 0.8246 | 0.8403 | 0.9275 |
| PWY-5705 | allantoin degradation to glyoxylate III | 0.1638 | 0.8176 | 0.8412 | 0.9275 |
| PWY-5022 | 4-aminobutanoate degradation V | 0.149 | 0.8048 | 0.8532 | 0.9275 |
| PWY-7392 | taxadiene biosynthesis (engineered) | 0.1488 | 0.8105 | 0.8543 | 0.9275 |
| SO4ASSIM-PWY | sulfate reduction I (assimilatory) | 0.1398 | 0.476 | 0.7689 | 0.9228 |
| PWY-6470 | peptidoglycan biosynthesis V (&beta;-lactam resistance) | 0.1349 | 1.379 | 0.922 | 0.966 |
| HISDEG-PWY | L-histidine degradation I | 0.1121 | 0.8871 | 0.8995 | 0.9518 |
| PWY-6992 | 1,5-anhydrofructose degradation | 0.1007 | 0.7082 | 0.8869 | 0.9409 |
| PWY-7254 | TCA cycle VII (acetate-producers) | 0.09099 | 0.972 | 0.9254 | 0.9669 |
| PWY-7328 | superpathway of UDP-glucose-derived O-antigen building blocks biosynthesis | 0.08404 | 0.5037 | 0.8675 | 0.9346 |
| P162-PWY | L-glutamate degradation V (via hydroxyglutarate) | 0.05836 | 0.9726 | 0.9521 | 0.9774 |
| PWY-7031 | protein N-glycosylation (bacterial) | 0.04909 | 0.03395 | 0.1482 | 0.8503 |
| SALVADEHYPOX-PWY | adenosine nucleotides degradation II | 0.04796 | 0.6409 | 0.9404 | 0.9702 |
| PWY-622 | starch biosynthesis | 0.04332 | 0.06824 | 0.5256 | 0.8503 |
| P23-PWY | reductive TCA cycle I | 0.03844 | 0.6994 | 0.9562 | 0.9774 |
| PWY-722 | nicotinate degradation I | 0.03282 | 0.05634 | 0.5603 | 0.8503 |
| GOLPDLCAT-PWY | superpathway of glycerol degradation to 1,3-propanediol | 0.03148 | 1.471 | 0.9829 | 0.9867 |
| PWY-3801 | sucrose degradation II (sucrose synthase) | 0.03078 | 0.0469 | 0.5116 | 0.8503 |
| METHANOGENESIS-PWY | methanogenesis from H2 and CO2 | 0.02943 | 0.06794 | 0.6648 | 0.9052 |
| PWY-6143 | CMP-pseudaminate biosynthesis | 0.02305 | 0.02953 | 0.4352 | 0.8503 |
| PWY-6167 | flavin biosynthesis II (archaea) | 0.01827 | 0.08003 | 0.8194 | 0.9232 |
| PWY-7084 | nitrifier denitrification | 0.01536 | 0.0311 | 0.6213 | 0.8719 |
| PWY-6397 | mycolyl-arabinogalactan-peptidoglycan complex biosynthesis | 0.01407 | 0.7161 | 0.9843 | 0.9867 |
| PWY-6141 | archaetidylserine and archaetidylethanolamine biosynthesis | 0.01123 | 0.03498 | 0.7483 | 0.92 |
| PWY-1422 | vitamin E biosynthesis (tocopherols) | 0.00577 | 0.009882 | 0.5593 | 0.8503 |
| PWY-2221 | Entner-Doudoroff pathway III (semi-phosphorylative) | 0.005283 | 0.01012 | 0.6016 | 0.8671 |
| LIPASYN-PWY | phospholipases | 0.003035 | 0.006649 | 0.6481 | 0.8921 |
| PWY-7644 | heparin degradation | 0.00298 | 0.008823 | 0.7355 | 0.9196 |
| PWY-6562 | norspermidine biosynthesis | -0.00262 | 0.6412 | 0.9967 | 0.9967 |
| PWY-3081 | L-lysine biosynthesis V | -0.01013 | 0.01107 | 0.3601 | 0.8503 |
| PWY-6581 | spirilloxanthin and 2,2'-diketo-spirilloxanthin biosynthesis | -0.0107 | 0.01372 | 0.4355 | 0.8503 |
| PWY-6565 | superpathway of polyamine biosynthesis III | -0.02125 | 0.01562 | 0.1738 | 0.8503 |
| PWY-5656 | mannosylglycerate biosynthesis I | -0.02187 | 0.0367 | 0.5513 | 0.8503 |
| SULFATE-CYS-PWY | superpathway of sulfate assimilation and cysteine biosynthesis | -0.02325 | 0.4247 | 0.9563 | 0.9774 |
| PWY-3941 | &beta;-alanine biosynthesis II | -0.02716 | 0.03902 | 0.4864 | 0.8503 |
| PWY-7286 | 7-(3-amino-3-carboxypropyl)-wyosine biosynthesis | -0.03447 | 1.37 | 0.9799 | 0.9867 |
| PWY-6654 | phosphopantothenate biosynthesis III | -0.03454 | 1.37 | 0.9799 | 0.9867 |
| PWY-6148 | tetrahydromethanopterin biosynthesis | -0.03472 | 1.369 | 0.9798 | 0.9867 |
| PWY-7209 | superpathway of pyrimidine ribonucleosides degradation | -0.03493 | 0.08774 | 0.6906 | 0.9145 |
| P164-PWY | purine nucleobases degradation I (anaerobic) | -0.03705 | 0.4545 | 0.935 | 0.9698 |
| PWY-6142 | gluconeogenesis II (Methanobacterium thermoautotrophicum) | -0.04074 | 0.04178 | 0.3295 | 0.8503 |
| FUCCAT-PWY | fucose degradation | -0.04138 | 0.572 | 0.9423 | 0.9702 |
| PWY-7013 | L-1,2-propanediol degradation | -0.04652 | 1.307 | 0.9716 | 0.9858 |
| PWY-6174 | mevalonate pathway II (archaea) | -0.04846 | 1.165 | 0.9668 | 0.985 |
| PWY-7385 | 1,3-propanediol biosynthesis (engineered) | -0.05077 | 0.08148 | 0.5332 | 0.8503 |
| PWY-6519 | 8-amino-7-oxononanoate biosynthesis I | -0.05201 | 0.4398 | 0.9058 | 0.9562 |
| PWY-6350 | archaetidylinositol biosynthesis | -0.05292 | 1.339 | 0.9685 | 0.985 |
| PWY-7007 | methyl ketone biosynthesis | -0.06096 | 0.7914 | 0.9386 | 0.9702 |
| BIOTIN-BIOSYNTHESIS-PWY | biotin biosynthesis I | -0.06379 | 0.4078 | 0.8757 | 0.9387 |
| PWY-6608 | guanosine nucleotides degradation III | -0.07229 | 0.4892 | 0.8825 | 0.9409 |
| PWY-7527 | L-methionine salvage cycle III | -0.07556 | 0.4835 | 0.8758 | 0.9387 |
| PWY-6948 | sitosterol degradation to androstenedione | -0.07579 | 0.07748 | 0.3279 | 0.8503 |
| PWY-6731 | starch degradation III | -0.08139 | 0.05684 | 0.1521 | 0.8503 |
| PWY-5392 | reductive TCA cycle II | -0.08389 | 0.1621 | 0.6048 | 0.8687 |
| P562-PWY | myo-inositol degradation I | -0.08393 | 0.7687 | 0.9131 | 0.9614 |
| PWY0-41 | allantoin degradation IV (anaerobic) | -0.08479 | 1.031 | 0.9345 | 0.9698 |
| PWY-5507 | adenosylcobalamin biosynthesis I (early cobalt insertion) | -0.08623 | 0.4782 | 0.8569 | 0.9279 |
| PWY-4361 | S-methyl-5-thio-&alpha;-D-ribose 1-phosphate degradation | -0.0867 | 0.5082 | 0.8645 | 0.9338 |
| PWY-6353 | purine nucleotides degradation II (aerobic) | -0.09465 | 0.4898 | 0.8468 | 0.9275 |
| PWY-7391 | isoprene biosynthesis II (engineered) | -0.1135 | 1.157 | 0.9218 | 0.966 |
| PWY0-862 | (5Z)-dodec-5-enoate biosynthesis | -0.1191 | 0.5114 | 0.8159 | 0.9228 |
| FUC-RHAMCAT-PWY | superpathway of fucose and rhamnose degradation | -0.1192 | 0.4884 | 0.8072 | 0.9228 |
| PWY-6349 | CDP-archaeol biosynthesis | -0.1228 | 1.351 | 0.9276 | 0.9669 |
| PWY-7234 | inosine-5'-phosphate biosynthesis III | -0.1257 | 0.601 | 0.8344 | 0.9275 |
| PWYG-321 | mycolate biosynthesis | -0.1294 | 0.4861 | 0.7901 | 0.9228 |
| PWY-6282 | palmitoleate biosynthesis I (from (5Z)-dodec-5-enoate) | -0.1298 | 0.5284 | 0.806 | 0.9228 |
| PWY0-1061 | superpathway of L-alanine biosynthesis | -0.1299 | 0.4663 | 0.7806 | 0.9228 |
| PWY-5989 | stearate biosynthesis II (bacteria and plants) | -0.1308 | 0.5308 | 0.8054 | 0.9228 |
| GLYCOLYSIS-E-D | superpathway of glycolysis and Entner-Doudoroff | -0.1314 | 0.7008 | 0.8513 | 0.9275 |
| POLYAMSYN-PWY | superpathway of polyamine biosynthesis I | -0.1319 | 0.5659 | 0.8157 | 0.9228 |
| PWY-7211 | superpathway of pyrimidine deoxyribonucleotides de novo biosynthesis | -0.1325 | 0.3944 | 0.737 | 0.9196 |
| PWY-6404 | superpathway of mycolyl-arabinogalactan-peptidoglycan complex biosynthesis | -0.1344 | 0.1373 | 0.3275 | 0.8503 |
| PWY-6467 | Kdo transfer to lipid IVA III (Chlamydia) | -0.1377 | 0.5673 | 0.8082 | 0.9228 |
| PWY0-1261 | anhydromuropeptides recycling | -0.1388 | 0.4242 | 0.7435 | 0.92 |
| PWY-1269 | CMP-3-deoxy-D-manno-octulosonate biosynthesis I | -0.1413 | 0.5691 | 0.8039 | 0.9228 |
| FASYN-INITIAL-PWY | superpathway of fatty acid biosynthesis initiation (E. coli) | -0.142 | 0.5299 | 0.7888 | 0.9228 |
| PWY-7664 | oleate biosynthesis IV (anaerobic) | -0.1427 | 0.5055 | 0.7777 | 0.9228 |
| NAGLIPASYN-PWY | lipid IVA biosynthesis | -0.1587 | 0.579 | 0.784 | 0.9228 |
| PWY0-781 | aspartate superpathway | -0.1603 | 0.3815 | 0.6745 | 0.9124 |
| P108-PWY | pyruvate fermentation to propanoate I | -0.1618 | 0.6951 | 0.8159 | 0.9228 |
| PWY-7197 | pyrimidine deoxyribonucleotide phosphorylation | -0.1637 | 0.4191 | 0.696 | 0.9145 |
| PWY-7184 | pyrimidine deoxyribonucleotides de novo biosynthesis I | -0.1642 | 0.4002 | 0.6816 | 0.9132 |
| PWY-6383 | mono-trans, poly-cis decaprenyl phosphate biosynthesis | -0.1645 | 0.3401 | 0.6286 | 0.873 |
| PWY-5913 | TCA cycle VI (obligate autotrophs) | -0.1726 | 0.4599 | 0.7074 | 0.9145 |
| PWY-5910 | superpathway of geranylgeranyldiphosphate biosynthesis I (via mevalonate) | -0.1758 | 1.23 | 0.8863 | 0.9409 |
| PWY-922 | mevalonate pathway I | -0.177 | 1.237 | 0.8862 | 0.9409 |
| HSERMETANA-PWY | L-methionine biosynthesis III | -0.189 | 0.459 | 0.6805 | 0.9132 |
| PWY-7228 | superpathway of guanosine nucleotides de novo biosynthesis I | -0.1977 | 0.3986 | 0.6199 | 0.8719 |
| PRPP-PWY | superpathway of histidine, purine, and pyrimidine biosynthesis | -0.2023 | 0.365 | 0.5795 | 0.8529 |
| ARG+POLYAMINE-SYN | superpathway of arginine and polyamine biosynthesis | -0.2026 | 0.5021 | 0.6865 | 0.9145 |
| GLUCUROCAT-PWY | superpathway of &beta;-D-glucuronide and D-glucuronate degradation | -0.2333 | 0.3869 | 0.5464 | 0.8503 |
| PWY-5189 | tetrapyrrole biosynthesis II (from glycine) | -0.2347 | 0.4192 | 0.5756 | 0.8503 |
| FASYN-ELONG-PWY | fatty acid elongation -- saturated | -0.2379 | 0.4706 | 0.6132 | 0.8719 |
| PWY-7255 | ergothioneine biosynthesis I (bacteria) | -0.245 | 0.7867 | 0.7555 | 0.9215 |
| PWY-6125 | superpathway of guanosine nucleotides de novo biosynthesis II | -0.2479 | 0.3929 | 0.528 | 0.8503 |
| PWY-5154 | L-arginine biosynthesis III (via N-acetyl-L-citrulline) | -0.2525 | 0.5254 | 0.6308 | 0.8731 |
| PWY-5188 | tetrapyrrole biosynthesis I (from glutamate) | -0.2533 | 0.4035 | 0.5302 | 0.8503 |
| TCA | TCA cycle I (prokaryotic) | -0.2575 | 0.4157 | 0.5356 | 0.8503 |
| PENTOSE-P-PWY | pentose phosphate pathway | -0.2577 | 0.4605 | 0.5757 | 0.8503 |
| PWY-7237 | myo-, chiro- and scillo-inositol degradation | -0.2691 | 0.7151 | 0.7067 | 0.9145 |
| PWY-5198 | factor 420 biosynthesis | -0.2704 | 0.8385 | 0.7471 | 0.92 |
| P461-PWY | hexitol fermentation to lactate, formate, ethanol and acetate | -0.2756 | 0.8487 | 0.7454 | 0.92 |
| PWY-7196 | superpathway of pyrimidine ribonucleosides salvage | -0.2794 | 0.4107 | 0.4964 | 0.8503 |
| GALACT-GLUCUROCAT-PWY | superpathway of hexuronide and hexuronate degradation | -0.2877 | 0.4146 | 0.4876 | 0.8503 |
| THISYN-PWY | superpathway of thiamin diphosphate biosynthesis I | -0.2887 | 0.492 | 0.5574 | 0.8503 |
| FERMENTATION-PWY | mixed acid fermentation | -0.2921 | 0.3902 | 0.4541 | 0.8503 |
| HOMOSER-METSYN-PWY | L-methionine biosynthesis I | -0.2938 | 0.4437 | 0.508 | 0.8503 |
| PWY-6969 | TCA cycle V (2-oxoglutarate:ferredoxin oxidoreductase) | -0.2955 | 0.4486 | 0.5101 | 0.8503 |
| PWY0-162 | superpathway of pyrimidine ribonucleotides de novo biosynthesis | -0.2966 | 0.3991 | 0.4574 | 0.8503 |
| POLYAMINSYN3-PWY | superpathway of polyamine biosynthesis II | -0.3034 | 0.5659 | 0.5919 | 0.859 |
| PWY-6700 | queuosine biosynthesis | -0.3043 | 0.453 | 0.5018 | 0.8503 |
| MET-SAM-PWY | superpathway of S-adenosyl-L-methionine biosynthesis | -0.3105 | 0.4183 | 0.4579 | 0.8503 |
| PWY-841 | superpathway of purine nucleotides de novo biosynthesis I | -0.3142 | 0.3886 | 0.4188 | 0.8503 |
| POLYISOPRENSYN-PWY | polyisoprenoid biosynthesis (E. coli) | -0.317 | 0.4718 | 0.5017 | 0.8503 |
| ARGORNPROST-PWY | arginine, ornithine and proline interconversion | -0.3185 | 0.8882 | 0.7199 | 0.9196 |
| PWY0-166 | superpathway of pyrimidine deoxyribonucleotides de novo biosynthesis (E. coli) | -0.3194 | 0.3795 | 0.4001 | 0.8503 |
| PWY-7323 | superpathway of GDP-mannose-derived O-antigen building blocks biosynthesis | -0.3203 | 0.6452 | 0.6196 | 0.8719 |
| P42-PWY | incomplete reductive TCA cycle | -0.3232 | 0.5141 | 0.5296 | 0.8503 |
| PWY-7332 | superpathway of UDP-N-acetylglucosamine-derived O-antigen building blocks biosynthesis | -0.3294 | 1.242 | 0.7909 | 0.9228 |
| PWY-6703 | preQ0 biosynthesis | -0.3413 | 0.4486 | 0.4469 | 0.8503 |
| PWY-7090 | UDP-2,3-diacetamido-2,3-dideoxy-&alpha;-D-mannuronate biosynthesis | -0.3429 | 1.125 | 0.7604 | 0.9228 |
| PWY-5347 | superpathway of L-methionine biosynthesis (transsulfuration) | -0.3434 | 0.3999 | 0.3905 | 0.8503 |
| CRNFORCAT-PWY | creatinine degradation I | -0.3492 | 1.118 | 0.7548 | 0.9215 |
| DENOVOPURINE2-PWY | superpathway of purine nucleotides de novo biosynthesis II | -0.3493 | 0.3871 | 0.3668 | 0.8503 |
| COLANSYN-PWY | colanic acid building blocks biosynthesis | -0.3521 | 0.5883 | 0.5495 | 0.8503 |
| PWY-6901 | superpathway of glucose and xylose degradation | -0.3572 | 0.5261 | 0.4972 | 0.8503 |
| P4-PWY | superpathway of L-lysine, L-threonine and L-methionine biosynthesis I | -0.3582 | 0.3601 | 0.3199 | 0.8503 |
| PWY-6478 | GDP-D-glycero-&alpha;-D-manno-heptose biosynthesis | -0.3583 | 0.5437 | 0.5098 | 0.8503 |
| P441-PWY | superpathway of N-acetylneuraminate degradation | -0.3612 | 0.506 | 0.4753 | 0.8503 |
| P261-PWY | coenzyme M biosynthesis I | -0.3632 | 0.6592 | 0.5816 | 0.853 |
| PWY-7200 | superpathway of pyrimidine deoxyribonucleoside salvage | -0.3642 | 0.3929 | 0.354 | 0.8503 |
| PWY-5005 | biotin biosynthesis II | -0.379 | 1.506 | 0.8013 | 0.9228 |
| PWY-6147 | 6-hydroxymethyl-dihydropterin diphosphate biosynthesis I | -0.3878 | 0.4234 | 0.3598 | 0.8503 |
| PWY-6895 | superpathway of thiamin diphosphate biosynthesis II | -0.389 | 0.4136 | 0.3469 | 0.8503 |
| PWY-7187 | pyrimidine deoxyribonucleotides de novo biosynthesis II | -0.3936 | 0.3864 | 0.3084 | 0.8503 |
| PWY-7242 | D-fructuronate degradation | -0.4039 | 0.4741 | 0.3943 | 0.8503 |
| PANTO-PWY | phosphopantothenate biosynthesis I | -0.4142 | 0.4551 | 0.3629 | 0.8503 |
| PWY-6612 | superpathway of tetrahydrofolate biosynthesis | -0.4148 | 0.3947 | 0.2933 | 0.8503 |
| PWY-5695 | urate biosynthesis/inosine 5'-phosphate degradation | -0.4209 | 0.4006 | 0.2935 | 0.8503 |
| GALACTUROCAT-PWY | D-galacturonate degradation I | -0.4217 | 0.4689 | 0.3685 | 0.8503 |
| PWY-7539 | 6-hydroxymethyl-dihydropterin diphosphate biosynthesis III (Chlamydia) | -0.4267 | 0.4303 | 0.3214 | 0.8503 |
| PWY-6545 | pyrimidine deoxyribonucleotides de novo biosynthesis III | -0.4302 | 0.443 | 0.3316 | 0.8503 |
| PWY-5971 | palmitate biosynthesis II (bacteria and plants) | -0.4315 | 0.7382 | 0.5588 | 0.8503 |
| RHAMCAT-PWY | L-rhamnose degradation I | -0.4326 | 0.5599 | 0.4397 | 0.8503 |
| P241-PWY | coenzyme B biosynthesis | -0.4357 | 1.28 | 0.7336 | 0.9196 |
| PWY-6906 | chitin derivatives degradation | -0.4387 | 0.4742 | 0.3548 | 0.8503 |
| PWY-7210 | pyrimidine deoxyribonucleotides biosynthesis from CTP | -0.4394 | 0.586 | 0.4533 | 0.8503 |
| PWY-7198 | pyrimidine deoxyribonucleotides de novo biosynthesis IV | -0.4425 | 0.6197 | 0.4753 | 0.8503 |
| FOLSYN-PWY | superpathway of tetrahydrofolate biosynthesis and salvage | -0.4454 | 0.3976 | 0.2627 | 0.8503 |
| HEXITOLDEGSUPER-PWY | superpathway of hexitol degradation (bacteria) | -0.446 | 0.7106 | 0.5303 | 0.8503 |
| PWY-7663 | gondoate biosynthesis (anaerobic) | -0.4511 | 0.3961 | 0.2549 | 0.8503 |
| PWY-5973 | cis-vaccenate biosynthesis | -0.4557 | 0.3952 | 0.2488 | 0.8503 |
| PWY-6507 | 4-deoxy-L-threo-hex-4-enopyranuronate degradation | -0.4629 | 0.634 | 0.4653 | 0.8503 |
| PANTOSYN-PWY | pantothenate and coenzyme A biosynthesis I | -0.4681 | 0.4495 | 0.2977 | 0.8503 |
| GLUTORN-PWY | L-ornithine biosynthesis | -0.4704 | 0.4531 | 0.2992 | 0.8503 |
| PWY-5677 | succinate fermentation to butanoate | -0.4806 | 0.7185 | 0.5036 | 0.8503 |
| RIBOSYN2-PWY | flavin biosynthesis I (bacteria and plants) | -0.4825 | 0.4417 | 0.2747 | 0.8503 |
| PWY-5659 | GDP-mannose biosynthesis | -0.4848 | 0.5063 | 0.3383 | 0.8503 |
| GLUCONEO-PWY | gluconeogenesis I | -0.4861 | 0.4247 | 0.2524 | 0.8503 |
| PWY-6897 | thiamin salvage II | -0.4936 | 0.4614 | 0.2847 | 0.8503 |
| PWY-7220 | adenosine deoxyribonucleotides de novo biosynthesis II | -0.4949 | 0.3819 | 0.1951 | 0.8503 |
| PWY-7222 | guanosine deoxyribonucleotides de novo biosynthesis II | -0.4949 | 0.3819 | 0.1951 | 0.8503 |
| TRPSYN-PWY | L-tryptophan biosynthesis | -0.4994 | 0.4762 | 0.2943 | 0.8503 |
| PWY-7111 | pyruvate fermentation to isobutanol (engineered) | -0.5031 | 0.3961 | 0.204 | 0.8503 |
| PWY-6628 | superpathway of L-phenylalanine biosynthesis | -0.5041 | 0.4711 | 0.2845 | 0.8503 |
| PWY-6630 | superpathway of L-tyrosine biosynthesis | -0.5091 | 0.4685 | 0.2772 | 0.8503 |
| PWY-6892 | thiazole biosynthesis I (E. coli) | -0.5192 | 0.4788 | 0.2782 | 0.8503 |
| PYRIDNUCSYN-PWY | NAD biosynthesis I (from aspartate) | -0.5209 | 0.4473 | 0.2443 | 0.8503 |
| PWY-5484 | glycolysis II (from fructose 6-phosphate) | -0.5293 | 0.4435 | 0.2327 | 0.8503 |
| ARGSYNBSUB-PWY | L-arginine biosynthesis II (acetyl cycle) | -0.5309 | 0.4447 | 0.2326 | 0.8503 |
| PWY-6891 | thiazole biosynthesis II (Bacillus) | -0.5312 | 0.5299 | 0.3162 | 0.8503 |
| 1CMET2-PWY | N10-formyl-tetrahydrofolate biosynthesis | -0.5316 | 0.4194 | 0.2049 | 0.8503 |
| PWY0-1586 | peptidoglycan maturation (meso-diaminopimelate containing) | -0.5356 | 0.5238 | 0.3064 | 0.8503 |
| DAPLYSINESYN-PWY | L-lysine biosynthesis I | -0.5368 | 0.4153 | 0.1961 | 0.8503 |
| ASPASN-PWY | superpathway of L-aspartate and L-asparagine biosynthesis | -0.5369 | 0.5284 | 0.3096 | 0.8503 |
| PWY-6121 | 5-aminoimidazole ribonucleotide biosynthesis I | -0.5386 | 0.4201 | 0.1998 | 0.8503 |
| ILEUSYN-PWY | L-isoleucine biosynthesis I (from threonine) | -0.5434 | 0.4161 | 0.1915 | 0.8503 |
| VALSYN-PWY | L-valine biosynthesis | -0.5434 | 0.4161 | 0.1915 | 0.8503 |
| PWY-6126 | superpathway of adenosine nucleotides de novo biosynthesis II | -0.5437 | 0.4069 | 0.1815 | 0.8503 |
| PWY-5101 | L-isoleucine biosynthesis II | -0.5468 | 0.4214 | 0.1944 | 0.8503 |
| PWY-6122 | 5-aminoimidazole ribonucleotide biosynthesis II | -0.5469 | 0.4203 | 0.1933 | 0.8503 |
| PWY-6277 | superpathway of 5-aminoimidazole ribonucleotide biosynthesis | -0.5469 | 0.4203 | 0.1933 | 0.8503 |
| PWY-7400 | L-arginine biosynthesis IV (archaebacteria) | -0.5479 | 0.4422 | 0.2154 | 0.8503 |
| THRESYN-PWY | superpathway of L-threonine biosynthesis | -0.548 | 0.4297 | 0.2023 | 0.8503 |
| ARGSYN-PWY | L-arginine biosynthesis I (via L-ornithine) | -0.5484 | 0.4422 | 0.2149 | 0.8503 |
| PWY-7229 | superpathway of adenosine nucleotides de novo biosynthesis I | -0.5528 | 0.4117 | 0.1794 | 0.8503 |
| PWY-6163 | chorismate biosynthesis from 3-dehydroquinate | -0.5558 | 0.4299 | 0.1961 | 0.8503 |
| PWY-3001 | superpathway of L-isoleucine biosynthesis I | -0.5567 | 0.4245 | 0.1897 | 0.8503 |
| PWY-5304 | superpathway of sulfur oxidation (Acidianus ambivalens) | -0.5597 | 0.7615 | 0.4624 | 0.8503 |
| ARO-PWY | chorismate biosynthesis I | -0.5597 | 0.4262 | 0.1891 | 0.8503 |
| CALVIN-PWY | Calvin-Benson-Bassham cycle | -0.5611 | 0.413 | 0.1742 | 0.8503 |
| SER-GLYSYN-PWY | superpathway of L-serine and glycine biosynthesis I | -0.5624 | 0.4268 | 0.1876 | 0.8503 |
| COMPLETE-ARO-PWY | superpathway of aromatic amino acid biosynthesis | -0.5648 | 0.4267 | 0.1857 | 0.8503 |
| METH-ACETATE-PWY | methanogenesis from acetate | -0.5649 | 0.6908 | 0.4135 | 0.8503 |
| BRANCHED-CHAIN-AA-SYN-PWY | superpathway of branched amino acid biosynthesis | -0.5654 | 0.4218 | 0.1801 | 0.8503 |
| GLYCOLYSIS | glycolysis I (from glucose 6-phosphate) | -0.5706 | 0.424 | 0.1784 | 0.8503 |
| PWY-2942 | L-lysine biosynthesis III | -0.5719 | 0.4262 | 0.1797 | 0.8503 |
| PWY-5667 | CDP-diacylglycerol biosynthesis I | -0.5728 | 0.4097 | 0.1621 | 0.8503 |
| PWY0-1319 | CDP-diacylglycerol biosynthesis II | -0.5728 | 0.4097 | 0.1621 | 0.8503 |
| PWY-6123 | inosine-5'-phosphate biosynthesis I | -0.5732 | 0.4422 | 0.1949 | 0.8503 |
| PWY-7221 | guanosine ribonucleotides de novo biosynthesis | -0.5763 | 0.4289 | 0.1791 | 0.8503 |
| PWY-7219 | adenosine ribonucleotides de novo biosynthesis | -0.5783 | 0.4328 | 0.1815 | 0.8503 |
| NONMEVIPP-PWY | methylerythritol phosphate pathway I | -0.5793 | 0.446 | 0.1939 | 0.8503 |
| PWY-7560 | methylerythritol phosphate pathway II | -0.5793 | 0.446 | 0.1939 | 0.8503 |
| PWY-6387 | UDP-N-acetylmuramoyl-pentapeptide biosynthesis I (meso-diaminopimelate containing) | -0.58 | 0.4321 | 0.1795 | 0.8503 |
| PWY-5103 | L-isoleucine biosynthesis III | -0.5801 | 0.4278 | 0.1751 | 0.8503 |
| DTDPRHAMSYN-PWY | dTDP-L-rhamnose biosynthesis I | -0.5802 | 0.4606 | 0.2078 | 0.8503 |
| COA-PWY | coenzyme A biosynthesis I | -0.5811 | 0.4329 | 0.1795 | 0.8503 |
| HISTSYN-PWY | L-histidine biosynthesis | -0.5815 | 0.4441 | 0.1904 | 0.8503 |
| ANAEROFRUCAT-PWY | homolactic fermentation | -0.5828 | 0.4336 | 0.179 | 0.8503 |
| PEPTIDOGLYCANSYN-PWY | peptidoglycan biosynthesis I (meso-diaminopimelate containing) | -0.5833 | 0.433 | 0.1779 | 0.8503 |
| PWY-5097 | L-lysine biosynthesis VI | -0.5836 | 0.4351 | 0.1799 | 0.8503 |
| PWY-6385 | peptidoglycan biosynthesis III (mycobacteria) | -0.5841 | 0.4339 | 0.1783 | 0.8503 |
| PWY-7208 | superpathway of pyrimidine nucleobases salvage | -0.5845 | 0.4334 | 0.1775 | 0.8503 |
| PWY-6386 | UDP-N-acetylmuramoyl-pentapeptide biosynthesis II (lysine-containing) | -0.5856 | 0.4331 | 0.1764 | 0.8503 |
| TRNA-CHARGING-PWY | tRNA charging | -0.5864 | 0.4314 | 0.1741 | 0.8503 |
| PHOSLIPSYN-PWY | superpathway of phospholipid biosynthesis I (bacteria) | -0.5876 | 0.4236 | 0.1654 | 0.8503 |
| NONOXIPENT-PWY | pentose phosphate pathway (non-oxidative branch) | -0.5907 | 0.4307 | 0.1703 | 0.8503 |
| PWY-7377 | cob(II)yrinate a,c-diamide biosynthesis I (early cobalt insertion) | -0.5914 | 1.393 | 0.6713 | 0.911 |
| ANAGLYCOLYSIS-PWY | glycolysis III (from glucose) | -0.592 | 0.4348 | 0.1734 | 0.8503 |
| PWY-5686 | UMP biosynthesis | -0.5933 | 0.4357 | 0.1733 | 0.8503 |
| PWY-5121 | superpathway of geranylgeranyl diphosphate biosynthesis II (via MEP) | -0.6101 | 0.4553 | 0.1803 | 0.8503 |
| UDPNAGSYN-PWY | UDP-N-acetyl-D-glucosamine biosynthesis I | -0.6102 | 0.4337 | 0.1594 | 0.8503 |
| GLCMANNANAUT-PWY | superpathway of N-acetylglucosamine, N-acetylmannosamine and N-acetylneuraminate degradation | -0.6108 | 0.5822 | 0.2941 | 0.8503 |
| PWY-5104 | L-isoleucine biosynthesis IV | -0.6215 | 0.4665 | 0.1828 | 0.8503 |
| P221-PWY | octane oxidation | -0.6224 | 0.9298 | 0.5033 | 0.8503 |
| PWY-7199 | pyrimidine deoxyribonucleosides salvage | -0.6233 | 0.4691 | 0.1839 | 0.8503 |
| PWY-7456 | mannan degradation | -0.6282 | 0.8581 | 0.4641 | 0.8503 |
| OANTIGEN-PWY | O-antigen building blocks biosynthesis (E. coli) | -0.6282 | 0.4402 | 0.1536 | 0.8503 |
| PYRIDNUCSAL-PWY | NAD salvage pathway I | -0.6316 | 0.4744 | 0.1831 | 0.8503 |
| PWY4FS-7 | phosphatidylglycerol biosynthesis I (plastidic) | -0.6381 | 0.4329 | 0.1405 | 0.8503 |
| PWY4FS-8 | phosphatidylglycerol biosynthesis II (non-plastidic) | -0.6381 | 0.4329 | 0.1405 | 0.8503 |
| COBALSYN-PWY | adenosylcobalamin salvage from cobinamide I | -0.6468 | 0.4638 | 0.1631 | 0.8503 |
| GLYCOCAT-PWY | glycogen degradation I (bacterial) | -0.6609 | 0.4967 | 0.1833 | 0.8503 |
| PWY-6269 | adenosylcobalamin salvage from cobinamide II | -0.6626 | 0.472 | 0.1604 | 0.8503 |
| PWY-5509 | adenosylcobalamin biosynthesis from cobyrinate a,c-diamide I | -0.663 | 0.4725 | 0.1605 | 0.8503 |
| PWY-5676 | acetyl-CoA fermentation to butanoate II | -0.6665 | 0.5752 | 0.2466 | 0.8503 |
| PWY-6572 | chondroitin sulfate degradation I (bacterial) | -0.7052 | 1.9 | 0.7105 | 0.9145 |
| PWY-6151 | S-adenosyl-L-methionine cycle I | -0.707 | 0.4795 | 0.1403 | 0.8503 |
| PWY-6737 | starch degradation V | -0.7088 | 0.5235 | 0.1758 | 0.8503 |
| PWY-621 | sucrose degradation III (sucrose invertase) | -0.7099 | 0.5251 | 0.1764 | 0.8503 |
| PWY-6609 | adenine and adenosine salvage III | -0.717 | 0.4875 | 0.1413 | 0.8503 |
| RUMP-PWY | formaldehyde oxidation I | -0.7189 | 1.06 | 0.4978 | 0.8503 |
| PWY-5100 | pyruvate fermentation to acetate and lactate II | -0.7214 | 0.5044 | 0.1526 | 0.8503 |
| P122-PWY | heterolactic fermentation | -0.7234 | 1.119 | 0.5182 | 0.8503 |
| GLYCOGENSYNTH-PWY | glycogen biosynthesis I (from ADP-D-Glucose) | -0.7288 | 0.5103 | 0.1533 | 0.8503 |
| PWY0-1296 | purine ribonucleosides degradation | -0.7412 | 0.5003 | 0.1384 | 0.8503 |
| PWY0-1298 | superpathway of pyrimidine deoxyribonucleosides degradation | -0.743 | 0.5009 | 0.138 | 0.8503 |
| TEICHOICACID-PWY | teichoic acid (poly-glycerol) biosynthesis | -0.7445 | 0.6588 | 0.2584 | 0.8503 |
| PWY-6317 | galactose degradation I (Leloir pathway) | -0.761 | 0.5273 | 0.1489 | 0.8503 |
| P161-PWY | acetylene degradation | -0.7675 | 0.4939 | 0.1202 | 0.8503 |
| PWY0-1297 | superpathway of purine deoxyribonucleosides degradation | -0.7712 | 0.4846 | 0.1116 | 0.8503 |
| CODH-PWY | reductive acetyl coenzyme A pathway | -0.7775 | 1.604 | 0.6278 | 0.873 |
| PWY-5505 | L-glutamate and L-glutamine biosynthesis | -0.7836 | 0.6 | 0.1916 | 0.8503 |
| PWY-1861 | formaldehyde assimilation II (RuMP Cycle) | -0.7868 | 1.139 | 0.4896 | 0.8503 |
| P163-PWY | L-lysine fermentation to acetate and butanoate | -0.8095 | 1.193 | 0.4974 | 0.8503 |
| P124-PWY | Bifidobacterium shunt | -0.8111 | 1.154 | 0.4823 | 0.8503 |
| PWY-6588 | pyruvate fermentation to acetone | -0.8236 | 0.7889 | 0.2965 | 0.8503 |
| PWY-5265 | peptidoglycan biosynthesis II (staphylococci) | -0.8285 | 0.524 | 0.1139 | 0.8503 |
| PWY-5384 | sucrose degradation IV (sucrose phosphorylase) | -0.8425 | 0.591 | 0.154 | 0.8503 |
| PWY-5532 | adenosine nucleotides degradation IV | -0.9424 | 0.7137 | 0.1867 | 0.8503 |
| PWY-6471 | peptidoglycan biosynthesis IV (Enterococcus faecium) | -0.9526 | 0.6573 | 0.1473 | 0.8503 |
| PWY490-3 | nitrate reduction VI (assimilatory) | -0.9532 | 0.6378 | 0.1351 | 0.8503 |
| LACTOSECAT-PWY | lactose and galactose degradation I | -0.9669 | 1.044 | 0.3544 | 0.8503 |
| PWY-7371 | 1,4-dihydroxy-6-naphthoate biosynthesis II | -0.9679 | 1.978 | 0.6246 | 0.873 |
| PWY-6263 | superpathway of menaquinol-8 biosynthesis II | -0.9738 | 1.641 | 0.553 | 0.8503 |
| PWY-4722 | creatinine degradation II | -0.9778 | 0.9145 | 0.285 | 0.8503 |
| PWY-2941 | L-lysine biosynthesis II | -0.9896 | 0.844 | 0.241 | 0.8503 |
| PWY-7374 | 1,4-dihydroxy-6-naphthoate biosynthesis I | -1.044 | 0.6633 | 0.1155 | 0.8503 |
| LPSSYN-PWY | superpathway of lipopolysaccharide biosynthesis | -1.056 | 0.8068 | 0.1905 | 0.8503 |
| PWY-6339 | syringate degradation | -1.102 | 1.224 | 0.3678 | 0.8503 |
| PWY-6749 | CMP-legionaminate biosynthesis I | -1.196 | 0.9009 | 0.1845 | 0.8503 |
| PWY-7046 | 4-coumarate degradation (anaerobic) | -1.207 | 1.037 | 0.2442 | 0.8503 |
| PWY-5088 | L-glutamate degradation VIII (to propanoate) | -1.631 | 0.9174 | 0.07536 | 0.8503 |
| DHGLUCONATE-PYR-CAT-PWY | glucose degradation (oxidative) | -1.637 | 1.319 | 0.2146 | 0.8503 |
