# Supplementary material for: Gut microbiome in PCOS associates to serum metabolomics: a cross-sectional study
Source: Sci Rep. 2022 Dec 23;12:22184. doi: 10.1038/s41598-022-25041-4 (PMC9789036; doi:10.1038/s41598-022-25041-4)
Supplement: Supplementary file 5 — Supplementary Information 5. [file 41598_2022_25041_MOESM5_ESM.docx]

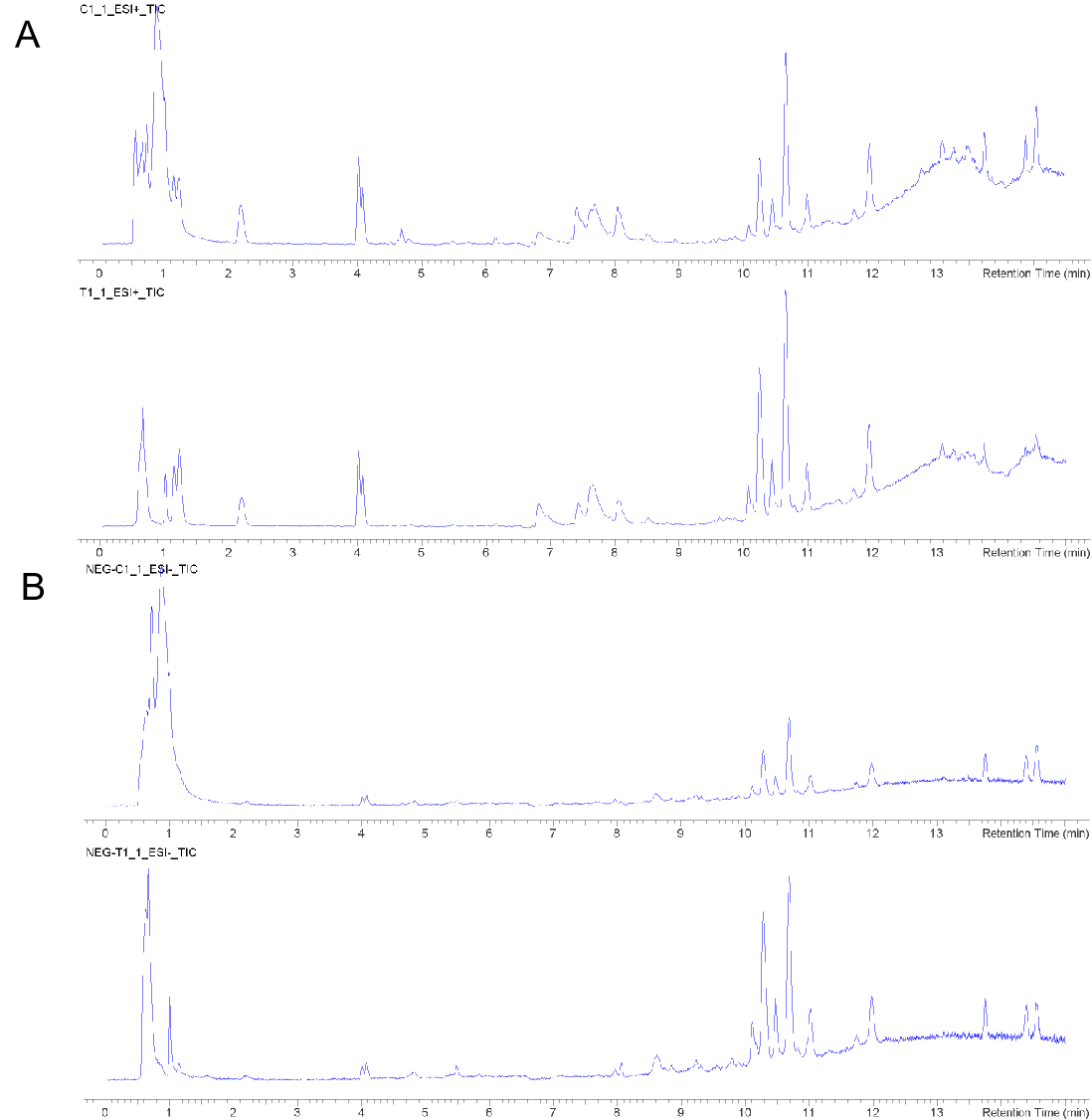


Figure 1 Total ion current chromatogram (TIC), with good reproducibility, indicating that the data results are reliable. Typical total ion chromatogram of the serum sample (pos) (A); typical total ion chromatogram of the serum sample (neg) (B)

The total ion current chromatograms (TIC) of the PCOS group and the control group in positive and negative ion modes are shown in Figure 1A-B. The TIC graphs (±) are stable and have good reproducibility, indicating that the results are reliable.
